# Clinical Landscape and Rate of Exposure to Ilheus Virus: Insights from Systematic Review and Meta-Analysis

**DOI:** 10.3390/v15010092

**Published:** 2022-12-29

**Authors:** Vivaldo Gomes da Costa, Marielena Vogel Saivish, Nikolas Alexander Borsato Lino, Cíntia Bittar, Marília de Freitas Calmon, Maurício Lacerda Nogueira, Paula Rahal

**Affiliations:** 1 Laboratório de Estudos Genômicos, Departamento de Biologia, Instituto de Biociências Letras e Ciências Exatas (IBILCE), Universidade Estadual Paulista (UNESP), São José do Rio Preto 15054-000, Brazil; 2 Laboratório de Pesquisas em Virologia, Departamento de Doenças Dermatológicas, Infecciosas e Parasitárias, Faculdade de Medicina de São José do Rio Preto (FAMERP), São José do Rio Preto 15090-000, Brazil

**Keywords:** Ilheus virus, arbovirus, neglected disease, meta-analysis, sero-epidemiology

## Abstract

Ilheus fever is a mosquito-borne, poorly known tropical disease. We aimed to report the pooled rate of exposure to the Ilheus virus (ILHV) and clinical outcomes of infection to determine the epidemiological patterns of ILHV. We conducted a meta-analysis of 37 studies (*n* = 17,722 individuals) from Latin America. The common clinical characteristics of ILHV infection were fever (82.3%), headache (52.9%), and myalgia (52.9%). Encephalitis complicated the course of the infection in 29.4% cases. Monotypic serological reactions detected a pooled rate of exposure of 2% to ILHV (95% CI: 1–2). Studies were mainly conducted in Brazil, with a pooled proportion of ILHV positivity of 8% (95% CI: 3–14). Males (12%) had higher rates of seropositivity than females (7%) and had high chances of ILHV infection (OR: 1.7, 95% CI: 1.2–2.5). Seropositivity increased with age, from 2% (95% CI: 2–3) among people aged 0–14 years to 8% (95% CI: 6–10) among people aged 15–64 years. Our analysis indicated a low and relatively constant burden of ILHV in Latin America. More research is needed to evaluate and innovate serological assays for ILHV to better estimate the burden and dynamics of epidemiological changes in ILHV infection in different regions.

## 1. Introduction

Ilheus virus (ILHV), a neglected human pathogen, is believed to be maintained in nature by an enzootic cycle between mosquito vectors and birds [1]. Structurally, ILHV has a spherical morphology with enveloped positive-sense single-stranded RNA and belongs to the genus *Flavivirus* in the Ntaya antigenic serocomplex [2,3]. It is phylogenetically associated with the Rocio virus (ROCV) with 73.37–74.5% amino acid identity, and ILHV and ROCV are considered members of the same species [4]. ILHV infection in humans can lead to Ilheus fever, an acute undifferentiated febrile disease with non-outbreak cases. However, the clinical spectrum can vary in severity, and the fever can progress to severe life-threatening meningoencephalitis [5,6,7,8,9,10]. In 2017, the first documented case of a fatality following ILHV detection via nucleic acid amplification in an elderly patient with encephalitis in Brazil drew public attention to the need to better understand the clinical–epidemiological scenario [11]. It is important to mention that in this case, it was not determined whether the virus caused encephalitis in the patient, who had co-morbidity (diabetes and hypertension).

Historically, ILHV was isolated in 1944 from a pool of *Psorophora* and *Aedes* mosquito species captured near Ilhéus city in Bahia state, Brazil, during an entomological surveillance of the yellow fever virus vector [1]. Viral infection in humans occurs via mosquito bites, especially from mosquitoes of the genera *Psorophora* and *Ochlerotatus*. ILHV was isolated from humans in 1959 in Belém city (Pará, Brazil). This was followed by viral isolation in Trinidad, West Indies (1962), Colombia (1966), Panama (1967), French Guiana (1973), Brazil (1995), and Ecuador (2007) [5,7,8,9,12,13,14]. Currently, several arboviral surveillance studies, including those on ILHV isolates, molecular identification, and serosurvey to detect antibodies against ILHV, have been carried out in the last few decades in South and Central America and in the Caribbean islands. Thus, according to the results of these studies, ILHV infections have been observed in several vertebrate species, such as monkeys, birds, rodents, bats, horses, coatis, and buffaloes (Supplementary Files S1 and S2) [15,16,17,18,19,20,21,22,23,24,25,26,27,28,29,30,31,32,33,34,35,36].

However, several barriers related to the difficulty in ILHV detection remain. The main factors include diverse endemic mosquito-borne flavivirus populations, a short viremic period, lack of specific clinical features, and the possibility of serological tests exhibiting cross-reactivity to shared epitopes on other endemic flaviviruses. Overall, these factors contribute to the gap in estimating the true ILHV burden on the population.

A clear and comprehensive understanding of the overall exposure rates to ILHV and its clinical manifestations is necessary to provide useful information for healthcare workers and policymakers and to underpin effective prevention and control strategies. However, these studies are lacking in scientific literature. The aims of this study were to compute the pooled rate of exposure to laboratory-confirmed cases of ILHV infection and describe the clinical characteristics of individuals following ILHV infection.

## 2. Materials and Methods

### 2.1. Systematic Review and Meta-Analysis

We performed a systematic review and meta-analysis according to the Preferred Reporting Items for Systematic reviews and Meta-Analysis (PRISMA) protocol (Supplementary File S3) [37]. The study protocol is available in PROSPERO with registration number CRD42022343010 (see https://www.crd.york.ac.uk/prospero/display_record.php?ID=CRD42022343010, accessed on 26 August 2022).

#### 2.1.1. Search Strategy

After defining the research protocol, we performed a systematic search in the PubMed, SciELO, and ScienceDirect databases. Articles in English, Spanish, and Portuguese were screened until July 2022, with no restrictions in terms of publication date. To refine the studies of interest, a combination of descriptors was used. We also identified additional studies by screening the reference lists of the selected articles and highly cited reviews of the topic of interest.

The following were the inclusion criteria: (1) original articles published in scientific journals containing information on serological and molecular surveys for the detection of ILHV in humans; (2) studies containing data related to the proportion and rate of viral infection by laboratory tests; (3) seroepidemiological surveys for the detection of anti-ILHV antibodies that included data concerning subgroups; and (4) demographic data from ILHV-positive patients for analyzing other factors, such as sex and age. The following exclusion criteria were adopted in our search strategy: (1) absence or confusing specification of the outcome of interest regarding ILHV positivity in laboratory tests; (2) revisions, book chapters, and seroprevalence studies not involving humans; and (3) small-scale seroepidemiological studies with sample sizes <15.

#### 2.1.2. Data Analysis

For all selected studies, we extracted the following data: first author, year of publication, place of study, baseline characteristics of study participants, including mean age, sex, method of diagnosis, monotypic or heterotypic serological reaction, number of humans investigated for ILHV infection, proportion of positive humans, and clinical signs and symptoms. The main outcomes of interest in the data analysis were: (1) the proportion of ILHV cases (laboratory-confirmed compared to clinically suspected ILHV cases); (2) the proportion of cases with recent and/or previous laboratory-confirmed ILHV infections among apparently healthy humans; and (3) the signs and symptoms reported in ILHV cases. The Newcastle–Ottawa Scale (NOS) was used to evaluate the risk of bias of the methodological quality of the included studies [38]. NOS evaluates group comparability, studies group selection, and verifies the exposure or outcome of interest in observational studies. NOS components were summed, and final scores were classified as: 0–2 low quality, 3 moderate quality, and 4–5 high quality [38].

#### 2.1.3. Statistical Analyses

Data were extracted using Microsoft Excel. Several tables containing dichotomous data (presence or absence of an event of interest) were generated for the relative and cumulative calculation of the frequencies of the outcomes of interest, with 95% CI. We performed a predefined subgroup analysis according to the origin and year of publication of the studies, sex and age of the participants, and serological assays used. The dichotomous data of the selected studies were extracted and plotted in a 2 × 2 table to obtain individual and combined odds ratios, with the purpose of analyzing risk factors (i.e., demographic characteristics: age, sex, and professional occupation) associated with ILHV positivity. Meta-analysis was performed using STATA IC/64 with the metaprop, metafunnel, and metaninf commands. The relative frequency of ILHV was determined by dividing the number of confirmed cases (ILHV positivity) by the pooled denominator (i.e., population), with results expressed as percentages. The variance of each frequency estimate (known as effect size (ES)) was calculated as *p*q/n, where *p* is the frequency, q is 1 − *p*, and n is the total number of humans screened [39]. Confidence intervals for the average ES were calculated using the following formula: 95% CI = ES ± 1.96 × SE, where SE is the standard error (SE = √(*p*q/n)). To ensure proportionate weight distribution to studies presenting extreme frequencies (near 0 or 1), we applied the Freeman–Tukey arcsine methodology [40,41,42]. Heterogeneity, that is, any kind of variability among the results of the studies, was assessed using the I^2^ index and Cochran’s Q test. I^2^ values of 25%, 50%, and >75% indicated low, moderate, and high heterogeneities among studies, respectively. Cochran’s Q test *p*-value < 0.05 was consistent with significant heterogeneity (I^2^ > 75%) [42]. Due to the nature of the studies, the existence of heterogeneity was expected; therefore, we chose to use the random-effects model for the meta-analysis, as proposed by DerSimonian-Laird [43]. We performed sensitivity analysis to test the effect of the influence of each study on the overall estimate. Furthermore, we performed subgroup analysis to reduce the possibility of heterogeneity. Publication bias was determined by the visual inspection of Begg’s funnel plot and Egger’s test calculations. For all tests, *p* values < 0.05 were considered statistically significant [44,45].

## 3. Results

### 3.1. Characteristics of the Included Studies and Quality Assessment

**Figure 1:**
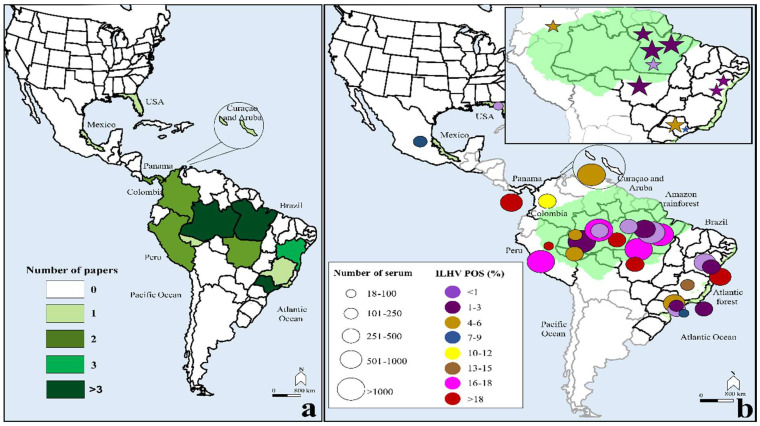
Map illustrating the geographical distribution of the included studies. (**a**) Color intensity represents the number of studies from each region. (**b**) Pooled estimated frequency of ILHV seropositivity. At the upper right of (**b**), the monotypic reaction data, which are samples that were only positive for ILHV (antibody against ILHV) and negative for at least one pair of tested flaviviruses, are shown.

We identified 308 studies using our initial search strategy (Supplementary File S4). After applying the inclusion and exclusion criteria, we were left with 37 studies that contributed to the systematic review and meta-analysis, all of which were conducted between 1962 and 2021 in the Americas (Supplementary Files S5 and S6) [5,6,7,8,9,10,11,12,13,14,36,46,47,48,49,50,51,52,53,54,55,56,57,58,59,60,61,62,63,64,65,66,67,68,69,70,71]. The median year of publication was 1975 (interquartile range [IQR] 1967–1997). The included studies reported 17,722 individuals, of which the majority came from the Amazon Region in Brazil (65.3%) [46,50,51,53,56,57,59,60,61,62,63,64,65,66,67,68,69,70,71], followed by Curaçao and Aruba (17%) [58], Peru (6.4%) [54,55], Panama (5.5%) [8,36], Colombia (3.8%) [12,52], Mexico (1.2%) [48], and the USA (0.1%) [47] (Figure 1). The sample sizes ranged from 18 to 3044 individuals. Regarding the study quality, evaluated by NOS, it was considered unlikely, as the mean score attained was moderate (3.6 score: Supplementary File S7).

There was a slight male predominance when stratified by sex (1.19:1) [9,36,50,53,55,60,61,62,63,65,66,68,70]. The general age profile of the individuals included in the studies was detailed in 9 (24.3%) of the 37 studies [7,9,36,46,53,58,62,63,70]. According to age category, there was representation in a ratio of 3:1:0.2 by age groups 15–64 years, <15 years, and >64 years, respectively.

**Figure 2:**
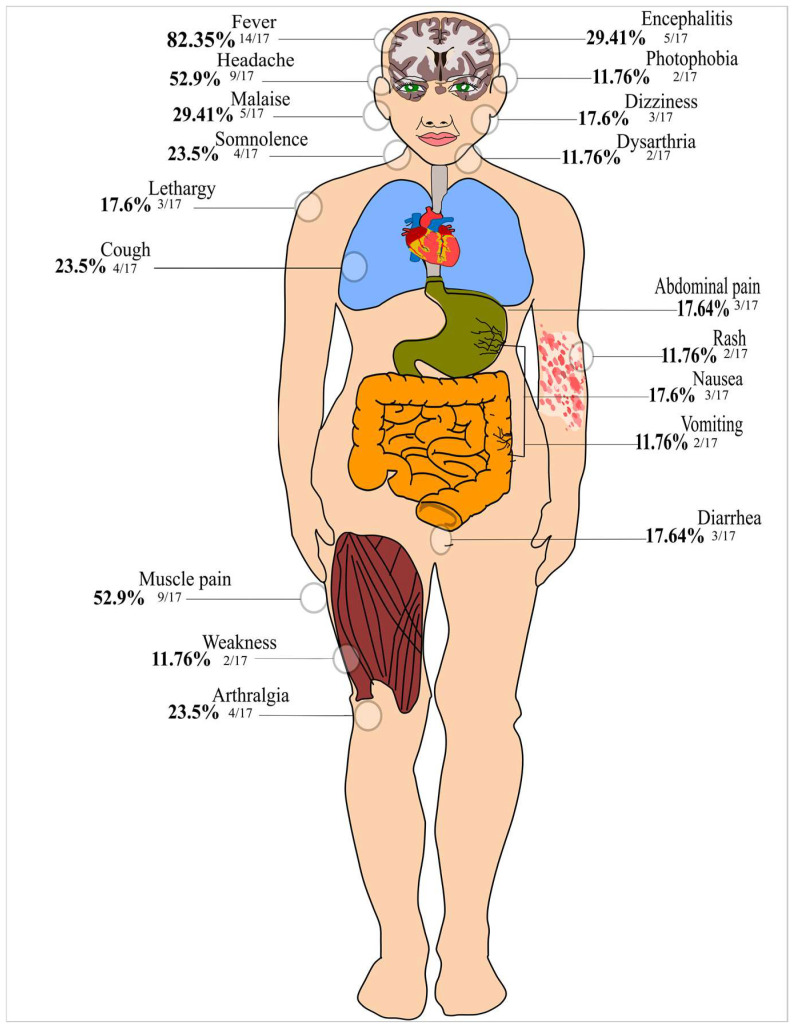
Clinical manifestations of ILHV infection [5,6,7,8,9,10,11,12,13,14].

Figure 2 depicts the clinical presentation of the individuals with ILHV infection. The most prevalent clinical signs and symptoms were fever, headache, muscle pain, malaise, and encephalitis.

### 3.2. Sensitivity Analysis and Publication Bias in the Meta-Analysis

When performing a sensitivity analysis to assess the weight of each individual study on the combined exposure rate to ILHV, through the removal of individual studies, no studies were found to significantly affect the combined positivity (S08 File). Additionally, subgroup analyses were performed; however, for most results, high heterogeneity was observed (I^2^ > 75%). It should be emphasized that these results were expected, given that in observational epidemiological studies, there is considerable diversity due to variations in study design, detection methodology, and epidemiological variations.

In the analysis of publication bias, asymmetry of the funnel plot was noted for ILHV positivity following subgroups: serological surveys and heterotypic vs. monotypic reactions. However, when analyzing the asymmetry by Egger’s test, the result was not considered statistically significant (t = 1.24, *p* = 0.22 [serological surveys], t = 1.13 and *p* = 0.27 [heterotypic], t = −0.2, and *p* = 0.8 [monotypic reaction]), indicating no small-study effects.

### 3.3. A Meta-Analysis to Estimate the Pooled Frequency of ILHV Infection

**Figure 3:**
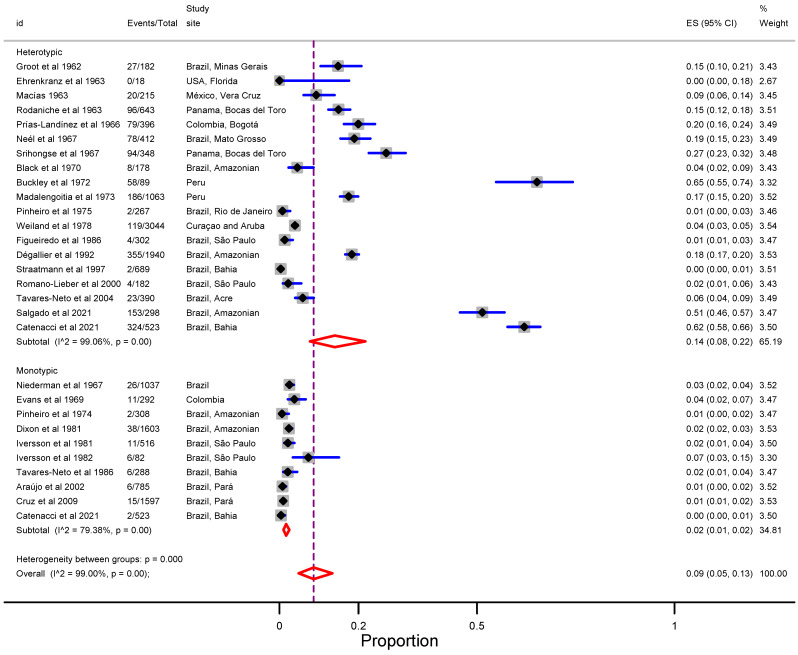
Forest plot of the proportion of laboratory confirmed ILHV infection according to the type of serological reaction (heterotypic vs monotypic) [8,36,46,47,48,49,50,51,52,53,54,55,56,57,58,59,60,61,62,63,64,65,66,67,68,69,70,71]. The black diamonds in gray squares indicate the mean of the ratio of ILHV positivity, while the size of the grey square represents the weight (population size) contributed by each study in the meta-analysis. The horizontal lines in blue represent their 95% confidence intervals (CI). The red diamond represents the pooled ratio of positives and its 95% CI, while dashed purple line represents the mean of the pooled estimate. id = identification of the study; Events = ILHV infection; CI = confidence interval; ES = effect size.

Hemagglutination inhibition (HI) and neutralization (NT) tests were used for the laboratory screening of anti-ILHV antibodies to confirm previous ILHV infections. The combined estimation of ILHV infection as detected through serological assays was 9% (95% CI: 5–13), with considerable evidence for regional epidemiological variation (Figure 3). The term monotypic serological reaction refers to a sample that was only positive for ILHV (i.e., antibody against ILHV) and negative for at least one pair of tested flaviviruses (i.e., Saint Louis encephalitis, yellow fever, dengue, Bussuquara, Rocio viruses). Thus, the results of heterotypic serological reactions (i.e., unspecified serological reaction if unique to ILHV) were higher than those of monotypic serological reactions (14%, 95% CI: 8–22 versus 2%, 95% CI: 1–2).

#### 3.3.1. Subgroup Analysis by Origin and Year of Publication of the Studies

In the analysis that evaluated seroprevalence stratified by region, the highest estimated pooled frequency (20%, 95% CI: 18–23, n = 1152 individuals) was observed in Peru. Panama (19%, 95% CI: 16–21, n = 991), Colombia (12%, 95% CI: 9–14, n = 689), Brazil (8%, 95% CI: 3–14, n = 11,587), and Curaçao and Aruba (4%, 95% CI: 3–5, n = 3044) contributed to the “top 5” regions in terms of ILHV seropositivity (Supplementary File S9).

**Figure 4:**
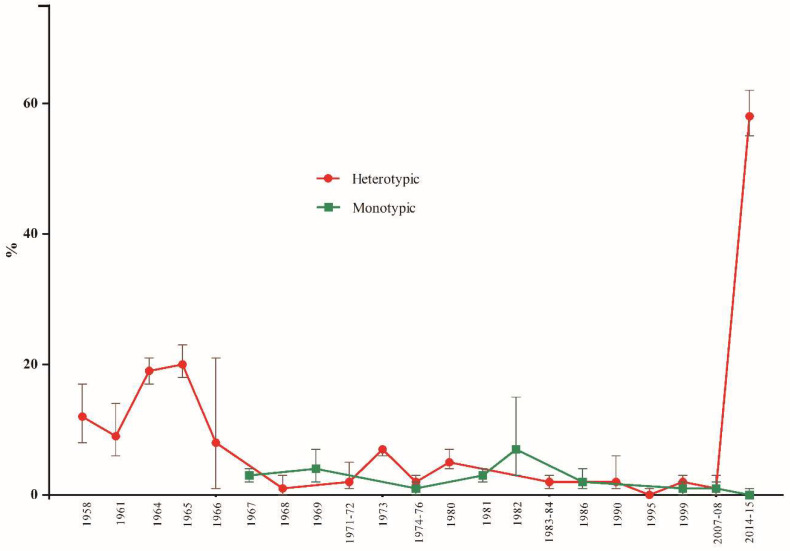
Distribution of ILHV seropositivity according to the year in which the samples were obtained. The values represent the mean with their respective 95% CI.

Seropositivity rates were observed to increase in studies performed in 2014–2015, although the general trend over the last few decades remains stable (Figure 4).

#### 3.3.2. Subgroup Analysis by Sex and Age of Participants, and Serological Assays Used

Children and adolescents (aged 0–14 years) had a lower frequency of ILHV infection (2%, 95% CI: 2–3, n = 3347 individuals) than individuals aged 15–64 years (8%, 95% CI: 6–10, n = 1374) (Supplementary File S10). Males had a higher pooled seroprevalence (12%, 95% CI = 10–14, n = 904) than females (7%, 95% CI = 5–9, n = 736).

Regarding the serological assays used, NT and HI had similar results, with overall ILHV positivities of 7% (95% CI: 2–15) and 9% (95% CI: 5–14), respectively.

#### 3.3.3. A Meta-Analysis to Evaluate Factors Associated with ILHV Positivity

Demographic characteristics (i.e., age, sex, and professional occupation) associated with ILHV occurrence were analyzed. Nonetheless, the only covariate available for testing in the analysis of risk factors for ILHV infection was the sex of infected participants. Males had significantly higher odds of seropositivity than females (OR = 1.78, 95% CI: 1.27–2.51) (Supplementary File S11).

## 4. Discussion

To the best of our knowledge, this is the first meta-analysis of ILHV infection. The main finding of the rates of ILHV seropositivity (9%, 95% CI: 5–13) included 17,722 individuals, with serological samples collected over many decades (1958–2015). Even when considering only the monotypic reactions, positivity remained at a considerable rate of 2% (95% CI: 1–2), with an indication of ILHV prevalence beyond the Amazon region (including parts of Brazil, Colombia, and Peru), as there was also positivity in the Brazilian states of Bahia and São Paulo. The moderate prevalence of ILHV-neutralizing antibodies, coupled with the low number of clinical cases of ILHV infection, suggests that most infections are inapparent or mild, which may be an important vehicle for viral spread to non-endemic regions [2,72,73].

Through our meta-analysis, we were able to elucidate the clinical characteristics of ILHV infection. Fever, headache, myalgia, encephalitis, malaise, arthralgia, gastrointestinal distress, and cough were the most frequently described clinical features. Since the infection is indistinguishable from other febrile syndromes and has a wide spectrum of clinical severity, ranging from subclinical to severe, a portion of symptomatic ILHV infections could be masked by clinical manifestations associated with multiple human pathogenic arboviruses [74], which may contribute to the underestimation of the burden of ILHV. Notably, no case of long-term sequelae or death has been linked to ILHV infection. This fact demands continuous efforts to establish effective public health arbovirus surveillance programs because although the number of confirmed cases of the disease is low, there is a high probability of underreporting due to difficulties in accurate laboratory diagnosis.

Regarding Figure 4, for the two studies from 2014–2015 [70,71], it is difficult to explain this high rate of seropositivity (mean = 58%, 95% CI: 55–62), but it is interesting to mention that these studies involved populations at high risk for arboviruses: male military personnel of working age in the Amazon rainforest or rural population. A high rate of heterotypic serological reactions were observed. These variables were also present in most of the other studies included in the meta-analysis; however, except for Buckley et al. [54], all other studies had lower ILHV positivity.

In South America, Central America, and the Caribbean, different geographic regions have multiple endemic mosquito-borne flaviviruses in co-circulation [75,76,77]. Twenty-nine (78.3%) of the 37 studies included in our meta-analysis investigated the antibody ratios against multiple arbovirus antigens. Flavivirus species have been linked to sporadic cases of human infection, including Bussuquara (BUSV), Cacipacore (CPCV), and ILHV [78,79]. All these viral pathogens may cause undifferentiated febrile illness in humans, and laboratory diagnosis is difficult due to the substantial cross-reactivity of antibodies [75]. Additionally, as occurs for ILHV, BUSV, CPCV, and other native flaviviruses are neglected, with misdiagnosis of cases compared to other medical arboviruses associated with outbreaks or epidemics that attract the most public attention, such as DENV, ZIKV, and YFV.

ILHV shares antigenic similarities with Bagaza, Ntaya, and Tembusu viruses in cross-neutralization tests [80]. These antigenic similarities are attributed to their genetic similarities. Interestingly, serocomplex cross-reactive memory can elicit viral cross-protection against infections with different serocomplexes [81,82]. In other words, polyclonal antisera (post-immune) against a specific virus can neutralize other viruses. As a result, Ilheus and Saint Louis encephalitis (SLE) flavivirus infections engender cross-protection against a lethal ROCV challenge in mice [83]. Similar cross-protection has also been demonstrated between Chikungunya and Mayaro alphaviruses and between DENV and West Nile and SLE flaviviruses [84,85]. New insights into protective ILHV infection responses are needed to elucidate its effects on previous flavivirus exposure and subsequent ILHV exposure. In addition, it is also important to establish whether ILHV immunity provides certain types of cross-protection against other co-circulating flaviviruses in certain geographic locations. Clarification of these factors is essential for a better understanding of the epidemiology of ILHV infection and for improving diagnostic methods.

Although seroepidemiological surveys are useful, this method could overestimate the frequency of ILHV positivity owing to cross-reactivity with other flavivirus infections. Since the included studies used HI/NT tests to capture total antibodies in the target serum, it was not possible to determine the dominant ILHV antigen (protein) involved. There is marked variation in the amino acid identity of the polyprotein of mosquito-borne flaviviruses; for example, in the polyprotein of Japanese encephalitis, dengue, yellow fever, Zika, and West Nile viruses, the amino acid similarity ranges between 45–77% [75]. As HI and serum neutralization tests target neutralizing antibodies, virus E (envelope) protein may be a promising target. The E protein shares 43–79% amino acid similarity between flaviviruses. The most cross-reactive ILHV antigen is the NS3 protein, which shares amino acid homology with the JEV complex [34]. In our meta-analysis, the cross-reactivity of serodiagnostic tests was likely modulated because we were able to demonstrate data on monotypic-type reactions [51,52,56,59,60,61,63,67,69,71].

The HI assay has been a mainstay serodiagnostic test for a long period of time, with the first assay described in the 1940s used to measure the hemagglutinating activity of the influenza virus [86]. Toward the end of the 1950s, antigens prepared from the Laemmert strain of ILHV were used in the HI assay [46]. The HI assay is highly applicable for the detection of anti-ILHV antibodies because no licensed kits are available for this purpose, and in-house HI presents low equipment and laboratory supply costs. A potential drawback is that this method is not commonly standardized, as is the plate-reduction neutralization test (PRNT) [87]. Thus, it is necessary to empirically determine how to weigh antibody cross-reactivity when interpreting the results. Therefore, new insights into ILHV serology assays that aid in determining the specificity (antibody cross-reactivity rate) and sensitivity across current and future laboratory techniques are of paramount importance.

A key factor to consider is the lack of detailed analyses of cross-neutralization studies involving ILHV cases; such studies may contribute to the level of uncertainty in ILHV diagnoses. This potential uncertainty may be mitigated by the studies included in our analysis (more than one-third) that used the viral neutralization assay, that is, NT, and showed good specificity [36,46,48,51,54,57,63,65,66,88,89]. Herein, in direct comparisons of NT and HI, similar results were noted, with overall ILHV positivities of 7% and 9%, respectively. Certainly, the search for neutralizing antibodies by PRNT, considered the “gold standard” in discriminating serodiagnosis of flavivirus infections, will contribute in reducing false-positive cases. However, PRNT may require handling live pathogens in a biosafety level 3–4 laboratory and is a labor-intensive and costly technique for routine laboratory use, making it difficult to be employed on a large scale [88,89].

Our study has important strengths, including the meta-analytic approach and the inclusion of 37 studies describing the serological and clinical characteristics of a large number of individuals. Although studies with small sample sizes were included, they did not impact the estimated pooled rate of exposure, likely because only three studies had sample sizes <100 individuals. The potential limitations of our study include the inconsistent availability of data in some of the selected studies. Several studies failed to report basic demographic details, such as sex, age, and professional activities, and were therefore excluded from subgroup analyses. Demographic variables are important as they enable the assessment of potential risk factors associated with higher seroprevalence. Further studies with complete data are required. Second, because most studies were not designed to determine the prevalence and incidence rates of ILHV infection, it was only possible to estimate the individual and combined proportions regarding the frequency of positivity of the pathogen. Therefore, future studies that are better distributed in the Americas and have data on monotypic serological reactions are necessary. A third potential limitation was the heterogeneity observed in the majority of the analyses, which was possibly due to differences in epidemiological settings, such as the endemicity of place linked with transmission intensity, differences in population groups with greater or lesser probability of exposure to the mosquito vector, laboratory accuracy of the techniques, and varied period of time.

## 5. Conclusions

In summary, our evidence suggests that due to the low and relatively constant positivity rate found for ILHV, research focused on the diagnosis of this virus should be a priority, especially for the detection of infrequent regional flaviviruses in undifferentiated febrile cases. As the majority of the studies included in our meta-analysis were carried out several decades ago and in concentrated locations, it is essential to encourage novel research, mainly comprising data on monotypic reactions, to trace a more accurate epidemiological pattern of ILHV infections. These studies are essential to understand the varying trends in ILHV epidemiology.

## Data Availability

Not applicable.

## Supplementary Materials

The supporting information can be downloaded at: https://www.mdpi.com/article/10.3390/v15010092/s1.
