# Supplementary material for: Clinical Landscape and Rate of Exposure to Ilheus Virus: Insights from Systematic Review and Meta-Analysis
Source: Viruses. 2022 Dec 29;15(1):92. doi: 10.3390/v15010092 (PMC9861323; doi:10.3390/v15010092)
Supplement: Supplementary file 1 [file viruses-15-00092-s001.zip › viruses-2073188-supplementary.pdf]

S1 File. Species of living beings with detected antibodies, RNA or positive isolates of ILHV.

**SPECIES isolation**	**STUDY**	**SPECIES Antibody/or RNA detection**	**STUDY**
		Columbina talpacoti (Bird), *Crotophaga ani*, *Amazilia versicolor*, *Dysithamnus mentalis*, *Myophobus fasciatus*, *Elaenia chiriquensis*, *Phyllomyias fasciatus*, *Leptopogon amaurocephalus*, *Pipromorpha rufiventris*, *Thryothorus longirostris*, *Troglodytes aedon*, *Turdus rufiventris*, *Turdus albicollis*, *Passer domesticus*, *Geothlypis aequinoctialis*, *Thraupis sayaca*, *Ramphocelus bresilius*, *Tachyphonus coronatus*, *Sporophila caerulescens*, *Zonotrichia capensis*	15. Ferreira, I.B.; Pereira, L.E.; Rocco, I.M.; Marti, A.T.; de Souza, L.T.; Iversson, L.B. Surveillance of arbovirus infections in the Atlantic forest region, state of São Paulo, Brazil. *Rev. Inst. Med. Trop*. São Paulo. **1994**, 36, 265-274. doi: 10.1590/s0036-46651994000300011.
Sporophila Caerulescens (birds) *Molothrus bonariensis*		*Columbina talpacoti*, *Geopelia cuneata*, *Molothrus bonariensis*, *Sicalis flaveola*, *Callithrix jacchus (saguis)*, *Callithrix penicillata (saguis)*, *Nasua nasua.(Quati)*	16. Pereira, L.E.; Suzuki, A.; Coimbra, T.L.; de Souza, R.P.; Chamelet, E.L. Arbovírus Ilheus em aves silvestres (Sporophila caerulescens e Molothrus bonariensis) [Ilheus arbovirus in wild birds (Sporophila caerulescens and Molothrus bonariensis)]. *Rev. Saude Publica*. **2001**, 35, 119-23. doi: 10.1590/S0034-89102001000200003.
		Bubalus bubalis (Motypic reactions)	17. Casseb, A.R.; Cruz, A.V.; Jesus, I.S.; Chiang, J.O.; Martins, L.C.; Silva, S.P.; Henriques, D.F.; Casseb, L.M.; Vas-concelos, P.F. Seroprevalence of flaviviruses antibodies in water buffaloes (Bubalus bubalis) in Brazilian Amazon. *J. Venom. Anim. Toxins. Incl. Trop. Dis*. **2014**, 20, 9. doi: 10.1186/1678-9199-20-9.
		Horse	18. Iversson, L.B.; Silva, R.A.; da Rosa, A.P.; Barros, V.L. Circulation of eastern equine encephalitis, western equine encephalitis, Ilhéus, Maguari and Tacaiuma viruses in equines of the Brazilian Pantanal, South America. *Rev. Inst. Med. Trop. Sao Paulo*. **1993**, 35, 355-9. doi: 10.1590/s0036-46651993000400009.
		Horse	19. Pauvolid-Corrêa, A.; Campos, Z.; Juliano, R.; Velez, J.; Nogueira, R.M.; Komar, N. Serological evidence of widespread circulation of West Nile virus and other flaviviruses in equines of the Pantanal, Brazil. *PLoS. Negl. Trop. Dis*. **2014**, 8, e2706. doi: 10.1371/journal.pntd.0002706.

		Bradypus variegatus (sloths)<br>*Choloepus hoffmanni* (sloths)	20. Medlin, S.; Deardorff, E.R.; Hanley, C.S.; Vergneau-Grosset, C.; Siudak-Campfield, A.; Dallwig, R.; da Rosa, A.T.; Tesh, R.B.; Martin, M.P.; Weaver. S.C.; et al. Serosurvey of selected arboviral pathogens in free-ranging, two-toed sloths (Choloepus hoffmanni) and three-toed sloths (Bradypus variegatus) in Costa Rica, 2005-07. *J. Wildl. Dis*. **2016**, 52, 883-892. doi: 10.7589/2015-02-040.
		Alouatta caraya (monkey)	21. Morales, M.A.; Fabbri, C.M.; Zunino, G.E.; Kowalewski, M.M.; Luppo, V.C.; Enría, D.A.; Levis, S.C.; Calderón, G.E. Detection of the mosquito-borne flaviviruses, West Nile, Dengue, Saint Louis Encephalitis, Ilheus, Bussuquara, and Yellow Fever in free-ranging black howlers (Alouatta caraya) of Northeastern Argentina. *PLoS. Negl. Trop. Dis*. **2017**, 10, e0005351. doi: 10.1371/journal.pntd.0005351.
		Leontopithecus chrysomelas (sagui)	22. Catenacci, L.S.; Ferreira, M.; Martins, L.C.; De Vleeschouwer, K.M.; Cassano, C.R.; Oliveira, L.C.; Canale, G.; Deem, S.L.; Tello, J.S.; Parker, P.; Vasconcelos, P.F.C.; Travassos da Rosa, E.S. Surveillance of Arboviruses in Primates and Sloths in the Atlantic Forest, Bahia, Brazil. *Ecohealth*. **2018**, 15, 777-791. doi: 10.1007/s10393-018-1361-2.
		Antibodies to ILHV was not detected (horses, other livestock, and wild birds)	23. Thompson, N.N.; Auguste, A.J.; Coombs, D.; Blitvich, B.J.; Carrington, C.V.; da Rosa, A.P.; Wang, E.; Chadee, D.D.; Drebot, M.A.; Tesh, R.B.; Weaver, S.C.; Adesiyun, A.A. Serological evidence of flaviviruses and alphaviruses in livestock and wildlife in Trinidad. *Vector Borne Zoonotic Dis*. **2012**, 12, 969-78. doi: 10.1089/vbz.2012.0959.
		Culex declarator (mosquito)/RNA detection<br>*Culex* (Melanoconion)<br>*Ochlerotatus serratus*	24. Vieira, C.J.D.S.P.; Andrade, C.D.; Kubiszeski, J.R.; Silva, D.J.F.D.; Barreto, E.S.; Massey, A.L.; Canale, G.R.; Ber-nardo, C.S.S.; Levi, T.; Peres, C.A.; Bronzoni, R.V.M. Detection of Ilheus virus in mosquitoes from southeast Amazon, Brazil. *Trans. R. Soc. Trop. Med. Hyg*. **2019**, 113, 424-427. doi: 10.1093/trstmh/trz031.
		Alouatta caraya (Monkey)	25. Almeida, M.A.B.; Santos, E.D.; Cardoso, J.D.C.; Noll, C.A.; Lima, M.M.; Silva, F.A.E.; Ferreira, M.S.; Martins, L.C.; Vasconcelos, P.F.D.C.; Bicca-Marques, J.C. Detection of antibodies against Icoaraci, Ilhéus, and Saint Louis Encephalitis arboviruses during yellow fever monitoring surveillance in non-human primates (Alouatta caraya)

			in southern Brazil. *J. Med. Primatol*. **2019**, 48, 211-217. doi: 10.1111/jmp.12417.
Aedes scapularis	26. Pauvolid-Corrêa, A.; Kenney, J.L.; Couto-Lima, D.; Campos, Z.M.; Schatzmayr, H.G.; Nogueira, R.M.; Brault, A.C.; Komar, N. Ilheus virus isolation in the Pantanal, west-central Brazil. *PLoS. Negl. Trop. Dis*. **2013**, 18, e2318. doi: 10.1371/journal.pntd.0002318.		
		Aedes aegypti<br>*Cx. quinquefasciatus*	27. da Silva Ferreira, R.; de Toni Aquino da Cruz, L.C.; de Souza, V.J.; da Silva Neves, N.A.; de Souza, V.C.; Filho, L.C.F.; da Silva Lemos, P.; de Lima, C.P.S.; Naveca, F.G.; Atanaka, M.; Nunes, M.R.T.; Slhessarenko, R.D. In-sect-specific viruses and arboviruses in adult male culicids from Midwestern Brazil. *Infect. Genet. Evol*. **2020**, 85, 104561. doi: 10.1016/j.meegid.2020.104561
		Anopheles triannulatus (RNA detection)<br>*Mansonia* spp.,<br>*Coquillettidia juxtamansonia*, Culex sp.	28. Cunha, M.S.; Luchs, A.; Dos Santos, F.C.P.; Caleiro, G.S.; Nogueira, M.L.; Maiorka, P.C. Applying a pan-flavivirus RT-qPCR assay in Brazilian public health surveillance. *Arch. Virol*. **2020**, 165, 1863-1868. doi: 10.1007/s00705-020-04680-w.
		Cx. (Mel.) *portesi*	29. Araújo, P.A.; Freitas, M.O.; Chiang, J.O.; Silva, F.A.; Chagas, L.L.; Casseb, S.M.; Silva, S.P.; Nunes-Neto, J.P.; Rosa-Júnior, J.W.; Nascimento, B.S.; et al. Investigation about the Occurrence of Transmission Cycles of Arbovirus in the Tropical Forest, Amazon Region. *Viruses*. **2019**, 11, 774. doi: 10.3390/v11090774.
		Culex sp., (RNA detection)<br>*Anopheles triannulatus*,<br>*Coquillettidia juxtamansonia*	30. Cunha, M.S.; Luchs, A.; da Costa, A.C.; Ribeiro, G.O.; Dos Santos, F.C.P.; Nogueira, J.S.; Komninakis, S.V.; Ma-rinho, R.D.S.S.; Witkin, S.S.; Villanova, F., et al. Detection and characterization of Ilheus and Iguape virus genomes in historical mosquito samples from Southern Brazil. *Acta Trop*. **2020**, 205, 105401. doi: 10.1016/j.actatropica.2020.105401.
Sabethes chloropterus	31. De Rodaniche, E.; Galindo, P. Isolation of Ilhéus virus from Sabethes chloropterus captured in Guatemala in 1956. *Am. J. Trop. Med. Hyg*. **1957**, 6, 686-7. doi: 10.4269/ajtmh.1957.6.686..		

Haemagogus spegazzinii<br>genus *Trichoprosopon*	32. De Rodaniche, E.; Galindo, P. Isolation of the virus of IIheus encephalitis from mosquitoes captured in Panama. *Am. J. Trop. Med. Hyg.* **1961**, 10, 393-4. doi: 10.4269/ajtmh.1961.10.393.		
genus *Psorophora*	33. De Rodaniche, E. Isolation of the virus of IIhéus encephalitis from mosquitoes of the genus Psorophora captured in Honduras. *Am. J. Trop. Med. Hyg.* **1956**, 5, 797-801. doi: 10.4269/ajtmh.1956.5.797.		
-	34. Aitken, T.H.; Anderson, C.R.; Downs, W.G. The isolation of Ilhéus virus from wild caught forest mosquitoes in Trinidad. *Am. J. Trop. Med. Hyg.* **1956**, 5, 621-5. doi: 10.4269/ajtmh.1956.5.621.		
	35. Galindo, P.; de Rodaniche, E. Birds as hosts of Ilheus encephalitis virus in Panama. Am. J. Trop. Med. Hyg. **1961**, 10, 395-6. doi: 10.4269/ajtmh.1961.10.395.		
Psorophora lutzii, P. *ferox*, *Culex nigripalpus* and *Aedes angustivittatus*<br>*Ramphocelus passerinii* (bird)<br>lower mammals	36. Rodaniche, Ed; Galindo, P. Ecological Observations on Ilhéus Virus in the Vicinity of Almirante, Republic of Panama*. *Am J. Trop. Med. Hyg.* **1963**, 12, 924-928, doi: 10.4269/ajtmh.1963.12.924.		

S2 File. Geographic distribution of ILHV-infection in non-human hosts.

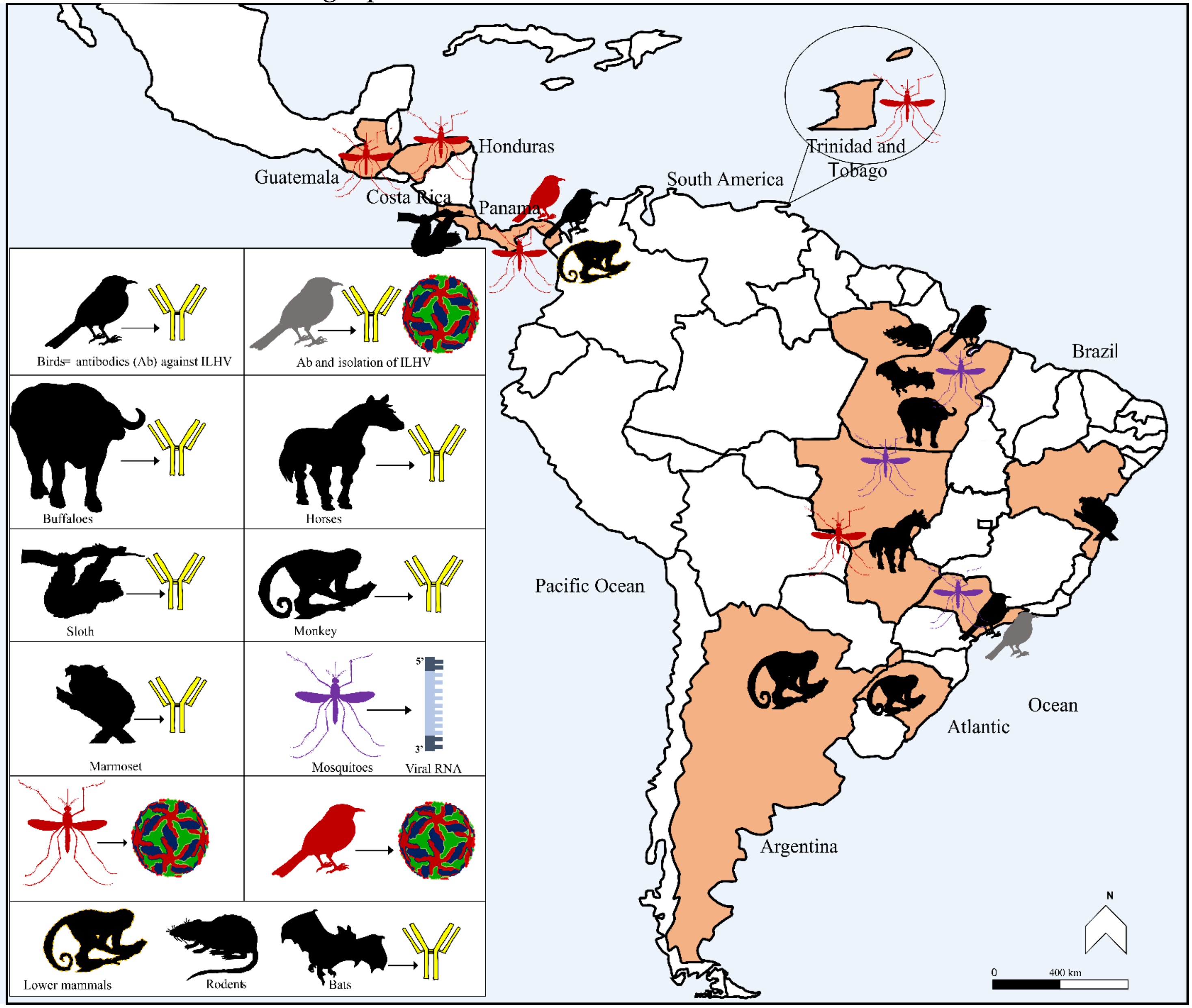

S3 File. PRISMA 2009 checklist.

Section/topic	#	Checklist item	Reported on page #
**TITLE**			
Title	1	Identify the report as a systematic review, meta-analysis, or both.	01
**ABSTRACT**			
Structured summary	2	Provide a structured summary including, as applicable: background; objectives; data sources; study eligibility criteria, participants, and interventions; study appraisal and synthesis methods; results; limitations; conclusions and implications of key findings; systematic review registration number.	01
**INTRODUCTION**			
Rationale	3	Describe the rationale for the review in the context of what is already known.	02
Objectives	4	Provide an explicit statement of questions being addressed with reference to participants, interventions, comparisons, outcomes, and study design (PICOS).	03
**METHODS**			
Protocol and registration	5	Indicate if a review protocol exists, if and where it can be accessed (e.g., Web address), and, if available, provide registration information including registration number.	03
Eligibility criteria	6	Specify study characteristics (e.g., PICOS, length of follow-up) and report characteristics (e.g., years considered, language, publication status) used as criteria for eligibility, giving rationale.	03
Information sources	7	Describe all information sources (e.g., databases with dates of coverage, contact with study authors to identify additional studies) in the search and date last searched.	03
Search	8	Present full electronic search strategy for at least one database, including any limits used, such that it could be repeated.	03
Study selection	9	State the process for selecting studies (i.e., screening, eligibility, included in systematic review, and, if applicable, included in the meta-analysis).	03
Data collection process	10	Describe method of data extraction from reports (e.g., piloted forms, independently, in duplicate) and any processes for obtaining and confirming data from investigators.	03
Data items	11	List and define all variables for which data were sought (e.g., PICOS, funding sources) and any assumptions and simplifications made.	03
Risk of bias in individual studies	12	Describe methods used for assessing risk of bias of individual studies (including specification of whether this was done at the study or outcome level), and how this information is to be used in any data synthesis.	03
Summary measures	13	State the principal summary measures (e.g., risk ratio, difference in means).	03-04

Synthesis of results	14	Describe the methods of handling data and combining results of studies, if done, including measures of consistency (e.g., I^2) for each meta-analysis.	03-04

Section/topic	#	Checklist item	Reported on page #
Risk of bias across studies	15	Specify any assessment of risk of bias that may affect the cumulative evidence (e.g., publication bias, selective reporting within studies).	03-04
Additional analyses	16	Describe methods of additional analyses (e.g., sensitivity or subgroup analyses, meta-regression), if done, indicating which were pre-specified.	03-04
**RESULTS**			
Study selection	17	Give numbers of studies screened, assessed for eligibility, and included in the review, with reasons for exclusions at each stage, ideally with a flow diagram.	04-05
Study characteristics	18	For each study, present characteristics for which data were extracted (e.g., study size, PICOS, follow-up period) and provide the citations.	04-05
Risk of bias within studies	19	Present data on risk of bias of each study and, if available, any outcome level assessment (see item 12).	04
Results of individual studies	20	For all outcomes considered (benefits or harms), present, for each study: (a) simple summary data for each intervention group (b) effect estimates and confidence intervals, ideally with a forest plot.	04-07
Synthesis of results	21	Present results of each meta-analysis done, including confidence intervals and measures of consistency.	04-07
Risk of bias across studies	22	Present results of any assessment of risk of bias across studies (see Item 15).	07
Additional analysis	23	Give results of additional analyses, if done (e.g., sensitivity or subgroup analyses, meta-regression [see Item 16]).	04-07
**DISCUSSION**			
Summary of evidence	24	Summarize the main findings including the strength of evidence for each main outcome; consider their relevance to key groups (e.g., healthcare providers, users, and policy makers).	07-08
Limitations	25	Discuss limitations at study and outcome level (e.g., risk of bias), and at review-level (e.g., incomplete retrieval of identified research, reporting bias).	09
Conclusions	26	Provide a general interpretation of the results in the context of other evidence, and implications for future research.	09
**FUNDING**			
Funding	27	Describe sources of funding for the systematic review and other support (e.g., supply of data); role of funders for the systematic review.	09

From: Moher D, Liberati A, Tetzlaff J, Altman DG, The PRISMA Group (2009). Preferred Reporting Items for Systematic Reviews and Meta-Analyses: The PRISMA Statement. PLoS Med 6(7): e1000097. doi:10.1371/journal.pmed1000097

S4 File. List of articles obtained through the use of descriptors in selected databases.

Descriptors: 'Neglected arbovirus' OR 'Ilheus virus' OR 'Ilheus arbovirus' from 1944 to 2022

Research Results PubMed: 157 (Neglected arbovirus); SciELO: 11 (Ilheus virus); SCIENCEDIRECT: 83 (Ilhéus virus or Ilheus arbovirus) (original research/news/short communications /others). [Duplicate= 4]. Adittional records *n*= 57. *n* Total= 308

In green: selected paper (*n*=37)

In yellow: paper excluded in the last selection step (*n*=16)

In Bold: duplicate studies (*n*=4)

N°	TITLE	JUSTIFICATION FOR EXCLUSION	PERIODIC
	**PUBMED**		
01	Transcriptomic and small RNA response to Mayaro virus infection in Anopheles stephensi mosquitoes	Non-ILHV. Non-human.	PLoS Negl Trop Dis
02	Temporal and Spatiotemporal Arboviruses Forecasting by Machine Learning: A Systematic Review	Review.	Front Public Health
03	An Overview of Neglected Orthobunyaviruses in Brazil	Non-ILHV.	Viruses
04	Neglected tropical diseases in Australia: a narrative review	Non-ILHV.	Med J Aust
05	Arboviral disease record data - Dengue and Chikungunya, Brazil, 2013-2020	Dengue and Chikungunya viruses.	Sci Data
06	First evidence of pyrethroid resistance in Italian populations of West Nile virus vector Culex pipiens	West Nile virus. Non-human.	Med Vet Entomol
07	Development of Real-Time Molecular Assays for the Detection of Wesselsbron Virus in Africa	Non-ILHV.	Microorganisms
08	Computational vaccinology guided design of multi-epitope subunit vaccine against a neglected arbovirus of the Americas	Mayaro virus.	J Biomol Struct Dyn
09	Combination of GC-MS Molecular Networking and Larvicidal Effect against *Aedes aegypti* for the Discovery of Bioactive Substances in Commercial Essential Oils	Non-ILHV. Non-human.	Molecules
10	Current knowledge of vector-borne zoonotic pathogens in Zambia: A clarion call to scaling-up "One Health" research in the wake of emerging and re-emerging infectious diseases	Review.	PLoS Negl Trop Dis
11	The Impact of Deforestation, Urbanization, and Changing Land Use Patterns on the Ecology of Mosquito and Tick-Borne Diseases in Central America	Review.	Insects
12	Comparison of Different Mosquito Traps for Zoonotic Arbovirus Vectors in Papua New Guinea	Non-ILHV. Non-human.	Am J Trop Med Hyg
13	Machine learning and deep learning techniques to support clinical diagnosis of arboviral diseases: A systematic review	Review.	PLoS Negl Trop Dis
14	Evaluation of Antiviral Activity of Cyclic Ketones against Mayaro Virus	Mayaro virus.	Viruses
15	An update on the mosquito fauna and mosquito-borne diseases distribution in Cameroon	Non-ILHV.	Parasit Vectors
16	Antioxidant and antiviral activity of fullerol against Zika virus	Zika virus.	Acta Trop
17	Observational Characterization of the Ecological and Environmental Features Associated with the Presence of Oropouche Virus and the Primary Vector *Culicoides paraenesis*: Data Synthesis and Systematic Review	Review.	Trop Med Infect Dis

18	Pathology and One Health implications of fatal Leptospira interrogans infection in an urbanized, free-ranging, black-tufted marmoset (Callithrix penicillata) in Brazil	Non-arbovirus	Transbound Emerg Dis
19	Viral and Prion Infections Associated with Central Nervous System Syndromes in Brazil	Review	Viruses
20	Mother-to-Child Transmission of Arboviruses during Breastfeeding: From Epidemiology to Cellular Mechanisms	Review	Viruses
21	Public health messages on arboviruses transmitted by Aedes aegypti in Brazil	Non-ILHV. Non-human.	BMC Public Health
22	Neurological disease caused by Oropouche virus in northern Brazil: should it be included in the scope of clinical neurological diseases?	Non-ILHV.	J Neurovirol
23	Japanese Encephalitis in Small-Scale Pig Farming in Rural Cambodia: Pig Seroprevalence and Farmer Awareness	Non-ILHV.	Pathogens
24	Toscana Virus: Ten Years of Diagnostics in Portugal	Non-ILHV.	Acta Med Port
25	Negeviruses Reduce Replication of Alphaviruses during Coinfection	Non-ILHV. Non-human.	J Virol
26	Emerging and Re-emerging Infectious Diseases in the WHO Eastern Mediterranean Region, 2001-2018	Review	Int J Health Policy Manag
27	Isolation and Characterization of Wuxiang Virus from Sandflies Collected in Yangquan County, Shanxi Province, China	Non-ILHV. Non-human.	Vector Borne Zoonotic Dis
28	Repurposing Drugs for Mayaro Virus: Identification of EIDD-1931, Favipiravir and Suramin as Mayaro Virus Inhibitors	Mayaro virus.	Microorganisms
29	Dynamics of Transmission of Urban Arbovirus Dengue, Zika and Chikungunya in Southwestern Region of Bahia, Brazil	Non-ILHV.	An Acad Bras Cienc
30	Experimental Challenge of Sheep and Cattle with Dugbe Orthonairovirus, a Neglected African Arbovirus Distantly Related to CCHFV	Non-ILHV.	Viruses
31	Mayaro virus detection in patients from rural and urban areas in Trinidad and Tobago during the Chikungunya and Zika virus outbreaks	Mayaro and chikungunya viruses.	Pathog Glob Health
32	Emerging and Neglected Viruses of Zoonotic Importance in Croatia	Review	Pathogens
33	The use of mobile phones for the prevention and control of arboviral diseases: a scoping review	Review	BMC Public Health
34	Arboviruses in the Astrakhan region of Russia for 2018 season: The development of multiplex PCR assays and analysis of mosquitoes, ticks, and human blood sera	Non-ILHV.	Infect Genet Evol
35	A mosquito small RNA genomics resource reveals dynamic evolution and host responses to viruses and transposons	Non-ILHV. Non-human.	Genome Res
36	Rate of exposure to Mayaro virus (MAYV) in Brazil between 1955 and 2018: a systematic review and meta-analysis	Review	Arch Virol
37	Type I interferons are essential while type II interferon is dispensable for protection against St. Louis encephalitis virus infection in the mouse brain	Non-ILHV. Non-human.	Virulence
38	Establishment of COVID-19 testing laboratory in resource-limited settings: challenges and prospects reported from Ethiopia	Non-ILHV.	Glob Health Action
39	NTDs in the age of urbanization, climate change, and conflict: Karachi, Pakistan as a case study	Review.	PLoS Negl Trop Dis
40	Microbial interactions in the mosquito gut determine Serratia colonization and blood-feeding propensity	Non-ILHV. Non-human.	ISME J
41	The limited knowledge of placental damage due to neglected infections: ongoing problems in Latin America	Review.	Syst Biol Reprod Med
42	Ivermectin: repurposing a multipurpose drug for Venezuela's humanitarian crisis	Review.	Int J Antimicrob Agents

43	Measuring Alphavirus Fidelity Using Non-Infectious Virus Particles	Non-ILHV. Non-human.	Viruses
44	Exploiting insect-specific viruses as a novel strategy to control vector-borne disease	Review	Curr Opin Insect Sci
45	From Anonymous to Public Enemy: How Does a Mosquito Become a Feared Arbovirus Vector?	Review.	Pathogens
46	Novel Toscana Virus Reverse Genetics System Establishes NSs as an Antagonist of Type I Interferon Responses	Non-ILHV.	Viruses
47	Abundance and Updated Distribution of *Aedes aegypti* (Diptera: Culicidae) in Cabo Verde Archipelago: A Neglected Threat to Public Health	Non-ILHV. Non-human.	Int J Environ Res Public Health
48	Oropouche infection a neglected arbovirus in patients with acute febrile illness from the Peruvian coast	Non-ILHV	BMC Res Notes
49	Implementation of bamboo and monkey-pot traps for the sampling cavity-breeding mosquitoes in Darién, Panama	Non-ILHV. Non-human.	Acta Trop
50	An emerging public health threat: Mayaro virus increases its distribution in Peru	Mayaro virus.	Int J Infect Dis
51	Patterns of insecticide resistance in Aedes aegypti: meta-analyses of surveys in Latin America and the Caribbean	Review.	Pest Manag Sci
52	Broader Geographical Distribution of Toscana Virus in the Mediterranean Region Suggests the Existence of Larger Varieties of Sand Fly Vectors	Non-ILHV. Non-human.	Microorganisms
53	An update on Toscana virus distribution, genetics, medical and diagnostic aspects	Review.	Clin Microbiol Infect
54	Neglected Australian Arboviruses Associated With Undifferentiated Febrile Illnesses	Non-ILHV.	Front Microbiol
55	The Role of Emerging and Neglected Viruses in the Etiology of Hepatitis	Review.	Curr Infect Dis Rep
56	The rise or fall of neglected tropical diseases in East Asia Pacific	Review.	Acta Trop
57	Characterization of a Novel Tanay Virus Isolated From *Anopheles sinensis* Mosquitoes in Yunnan, China	Non-ILHV. Non-human.	Front Microbiol
58	No evidence of Zika, dengue, or chikungunya virus infection in field-caught mosquitoes from the Recife Metropolitan Region, Brazil, 2015	Non-ILHV.	Wellcome Open Res
59	*Culicoides* Biting Midges-Underestimated Vectors for Arboviruses of Public Health and Veterinary Importance	Review.	Viruses
60	A Functional Ubiquitin-Proteasome System is Required for Efficient Replication of New World Mayaro and Una Alphaviruses	Mayaro and Una viruses.	Viruses
61	Active Essential Oils and Their Components in Use against Neglected Diseases and Arboviruses	Review.	Oxid Med Cell Longev
62	The role of co-infection and swarm dynamics in arbovirus transmission	Review.	Virus Res
63	Heat shock protein 70 (Hsp70) mediates Zika virus entry, replication, and egress from host cells	Zika virus.	Emerg Microbes Infect
64	Undetected Chikungunya virus co-infections in a Brazilian region presenting hyper-endemic circulation of Dengue and Zika	Non-ILHV. Non-human.	J Clin Virol
65	Emergence of the East-Central-South-African genotype of Chikungunya virus in Brazil and the city of Rio de Janeiro may have occurred years before surveillance detection	Non-ILHV. Non-human.	Sci Rep
66	Development of an Enzyme-Linked Immunosorbent Assay To Detect Antibodies Targeting Recombinant Envelope Protein 2 of Mayaro Virus	Mayaro virus.	J Clin Microbiol
67	Arbovirus coinfection and co-transmission: A neglected public health concern?	Review.	PLoS Biol
68	Oropouche Virus-Associated Aseptic Meningoencephalitis, Southeastern Brazil	Oropouche virus.	Emerg Infect Dis
69	Ghana: Accelerating neglected tropical disease control in a setting of economic development	Non-ILHV.	PLoS Negl Trop Dis
70	The decline of dengue in the Americas in 2017: discussion of multiple hypotheses	Review.	Trop Med Int Health

71	Using physical contact heterogeneity and frequency to characterize dynamics of human exposure to nonhuman primate bodily fluids in central Africa	Non-ILHV.	PLoS Negl Trop Dis
72	Predicting Yellow Fever Through Species Distribution Modeling of Virus, Vector, and Monkeys	Non-ILHV. Non-human.	Ecohealth
73	Integrated Aedes management for the control of Aedes-borne diseases	Review.	PLoS Negl Trop Dis
74	Antibody responses to Zika virus proteins in pregnant and non-pregnant macaques	Zika virus. Non-human.	PLoS Negl Trop Dis
75	Chikungunya virus infection prevalence in Africa: a contemporaneous systematic review and meta-analysis	Review.	Public Health
76	Seasonal and interannual risks of dengue introduction from South-East Asia into China, 2005-2015	Non-ILHV. Dengue virus.	PLoS Negl Trop Dis
77	Neglected vector-borne bacterial diseases and arboviruses in the Mediterranean area	Non-ILHV.	New Microbes New Infect
78	The Neglect and Fast Spread of Some Arboviruses: A Note for Healthcare Providers in Nigeria	Review.	Diseases
79	Dengue Virus Inhibition Targets: A Review and Docking Study	Review.	Curr Trop Med Chem
80	Insects and the Transmission of Bacterial Agents	Review.	Microbiol Spectr
81	Confronting the Emerging Threat to Public Health in Northern Australia of Neglected Indigenous Arboviruses	Non-ILHV.	Trop Med Infect Dis
82	Ongoing and emerging arbovirus threats in Europe	Review.	J Clin Virol
83	The genome of the biting midge Culicoides sonorensis and gene expression analyses of vector competence for bluetongue virus	Non-ILHV. Non-human.	BMC Genomics
84	Antiviral activity of silymarin against Mayaro virus and protective effect in virus-induced oxidative stress	Mayaro virus.	Antiviral Res
85	Neuronal Degeneration in Mice Induced by an Epidemic Strain of Saint Louis Encephalitis Virus Isolated in Argentina	Non-ILHV.	Front Microbiol
86	Persistent Replication of a Chikungunya Virus Replicon in Human Cells Is Associated with Presence of Stable Cytoplasmic Granules Containing Nonstructural Protein 3	Non-ILHV.	J Virol
87	Dispersion and oviposition of Aedes albopictus in a Brazilian slum: Initial evidence of Asian tiger mosquito domiciliation in urban environments	Non-ILHV. Non-human.	PLoS One
88	Oropouche Fever: A Review	Review.	Viruses
89	Heterogeneity of clinical isolates of chikungunya virus and its impact on the responses of primary human fibroblast-like synoviocytes	Non-ILHV.	J Gen Virol
90	Improved tools and strategies for the prevention and control of arboviral diseases: A research-to-policy forum	Review.	PLoS Negl Trop Dis
91	Effect of land-use changes on the abundance, distribution, and host-seeking behavior of Aedes arbovirus vectors in oil palm-dominated landscapes, southeastern Côte d'Ivoire	Non-ILHV. Non-human.	PLoS One
92	Neglected Australian Arboviruses and Undifferentiated Febrile Illness: Addressing Public Health Challenges Arising From the 'Developing Northern Australia' Government Policy	Non-ILHV.	Front Microbiol
93	Impact of environmental factors on neglected emerging arboviral diseases	Non-ILHV.	PLoS Negl Trop Dis
94	Association between suspected Zika virus disease during pregnancy and giving birth to a newborn with congenital microcephaly: a matched case-control study	Non-ILHV.	BMC Res Notes
95	International workshop on insecticide resistance in vectors of arboviruses, December 2016, Rio de Janeiro, Brazil	Non-ILHV.	Parasit Vectors
96	Emergence of recombinant Mayaro virus strains from the Amazon basin	Mayaro virus. Non-ILHV.	Sci Rep
97	Illegal gold miners in French Guiana: a neglected population with poor health	Non-ILHV.	BMC Public Health

98	Urbanization is a main driver for the larval ecology of Aedes mosquitoes in arbovirus-endemic settings in south-eastern Côte d'Ivoire	Non-ILHV. Non-human.	PLoS Negl Trop Dis
99	Venezuela and its rising vector-borne neglected diseases	Non-ILHV.	PLoS Negl Trop Dis
100	Preparing clinicians for (re-)emerging arbovirus infectious diseases in Europe	Review.	Clin Microbiol Infect
101	Identification of sympatric cryptic species of Aedes albopictus subgroup in Vietnam: new perspectives in phylosymbiosis of insect vector	Non-ILHV. Non-human.	Parasit Vectors
102	International workshop on insecticide resistance in vectors of arboviruses, December 2016, Rio de Janeiro, Brazil	Non-ILHV. Non-human.	Parasit Vectors
103	Neglected Australian arboviruses: quam gravis?	Review.	Microbes Infect
104	Leptospirosis in French Guiana and the Guiana shield: Current knowledge in 2016	Non-ILHV.	Bull Soc Pathol Exot
105	Oxidative stress in Mayaro virus infection	Mayaro virus. Non-ILHV.	Virus Res
106	Molecular and clinical epidemiological surveillance of dengue virus in Paraíba, Northeast Brazil	Dengue virus. Non-ILHV.	Rev Soc Bras Med Trop
107	Global urbanization and the neglected tropical diseases	Non-ILHV.	PLoS Negl Trop Dis
108	Dengue infection in the nervous system: lessons learned for Zika and Chikungunya	Review.	Arq Neuropsiquiatr
109	Aedes-Borne Virus-Mosquito Interactions: Mass Spectrometry Strategies and Findings	Review.	Vector Borne Zoonotic Dis
110	Oropouche Virus: Clinical, Epidemiological, and Molecular Aspects of a Neglected Orthobunyavirus	Review.	Am J Trop Med Hyg
111	Tracking Insecticide Resistance in Mosquito Vectors of Arboviruses: The Worldwide Insecticide resistance Network (WIN)	Non-ILHV. Non-human.	PLoS Negl Trop Dis
112	A Tale of Two Flaviviruses: A Seroepidemiological Study of Dengue Virus and West Nile Virus Transmission in the Ouest and Sud-Est Departments of Haiti	Non-ILHV.	Am J Trop Med Hyg
113	Dengue and West Nile Virus Transmission in Children and Adults in Coastal Kenya	Non-ILHV.	Am J Trop Med Hyg
114	Mayaro fever in an HIV-infected patient suspected of having Chikungunya fever	Non-ILHV.	Rev Soc Bras Med Trop
115	Do neglected Australian arboviruses pose a global epidemic threat?	Non-ILHV.	Aust N Z J Public Health
116	In-silico screening for anti-Zika virus phytochemicals	Zika virus. Non-ILHV.	J Mol Graph Model
117	Spatial and Temporal Hot Spots of Aedes albopictus Abundance inside and outside a South European Metropolitan Area	Non-ILHV. Non-human.	PLoS Negl Trop Dis
118	Zika virus: history of a newly emerging arbovirus	Review.	Lancet Infect Dis
119	Serological evidence of rift valley fever virus among acute febrile patients in Southern Mozambique during and after the 2013 heavy rainfall and flooding: implication for the management of febrile illness	Non-ILHV.	Virol J
120	Zika virus and Zika fever	Review.	Virol Sin
121	Review: A neglected Flavivirus: an update on Zika virus in 2016 and the future direction of research	Review.	Neuropathol Appl Neurobiol
122	Reconstruction of the Evolutionary History and Dispersal of Usutu Virus, a Neglected Emerging Arbovirus in Europe and Africa	Non-ILHV.	mBio
123	Benefits of using heterologous polyclonal antibodies and potential applications to new and undertreated infectious pathogens	Review.	Vaccine
124	Dengue virus persists and replicates during storage of platelet and red blood cell units	Dengue virus. Non-ILHV.	Transfusion
125	Complete Genome Sequence of Mayaro Virus Imported from the Amazon Basin to São Paulo State, Brazil	Mayaro virus. Non-ILHV.	Genome Announc

126	Plant-mediated biosynthesis of nanoparticles as an emerging tool against mosquitoes of medical and veterinary importance: a review	Review.	Parasitol Res
127	Syndromic Approach to Arboviral Diagnostics for Global Travelers as a Basis for Infectious Disease Surveillance	Non-ILHV.	PLoS Negl Trop Dis
128	Phylogenetic analysis of Bunyamwera and Ngari viruses (family Bunyaviridae, genus Orthobunyavirus) isolated in Kenya	Non-ILHV.	Epidemiol Infect
129	Recombinase Polymerase Amplification Assay for Rapid Diagnostics of Dengue Infection	Dengue virus. Non-ILHV.	PLoS One
130	Whole genome phylogenetic investigation of a West Nile virus strain isolated from a tick sampled from livestock in north eastern Kenya	Non-ILHV.	Parasit Vectors
131	A perspective on targeting non-structural proteins to combat neglected tropical diseases: Dengue, West Nile and Chikungunya viruses	Review.	Eur J Med Chem
132	Current pipelines for neglected diseases	Non-ILHV.	PLoS Negl Trop Dis
133	Neglected tropical diseases in Central America and Panama: review of their prevalence, populations at risk and impact on regional development	Review.	Int J Parasitol
134	Neglected tropical diseases in Central America and Panama: review of their prevalence, populations at risk and impact on regional development	Non-ILHV. Non-human.	J Vector Ecol
135	Tropical Medicine in the Horse Latitudes	Review.	Curr Trop Med Rep
136	Toscana virus infections: a case series from France	Non-ILHV.	J Infect
137	Acute disseminated encephalomyelitis associated with dengue infection: a case report with literature review	Review.	J Neurol Sci
138	The mosquito borne West Nile virus infection: is it threating to Egypt or a neglected endemic disease?	Non-ILHV.	J Egypt Soc Parasitol
139	Diversity of Culex torrentium Martini, 1925 - a potential vector of arboviruses and filaria in Europe	Non-ILHV. Non-human.	Parasitol Res
140	High content screening of a kinase-focused library reveals compounds broadly-active against dengue viruses	Non-ILHV. Non-human.	PLoS Negl Trop Dis
141	Biology of phlebotomine sand flies as vectors of disease agents	Review.	Annu Rev Entomol
142	Neglected infections of poverty in Texas and the rest of the United States: management and treatment options	Non-ILHV.	Clin Pharmacol Ther
143	New introduction and spread of rabies among dog population in Bangui	Non-ILHV. Non-human.	Acta Trop
144	Rift Valley fever virus(Bunyaviridae: Phlebovirus): an update on pathogenesis, molecular epidemiology, vectors, diagnostics and prevention	Non-ILHV.	Vet Res
145	Screening mosquito house entry points as a potential method for integrated control of endophagic filariasis, arbovirus and malaria vectors	Non-ILHV.	PLoS Negl Trop Dis
146	The neglected arboviral infections in mainland China	Review.	PLoS Negl Trop Dis
147	Biogeography of tick-borne bhanja virus (bunyaviridae) in europe	Non-ILHV.	Interdiscip Perspect Infect Dis
148	What comes after bluetongue--Europe as target for exotic arboviruses	Review.	Berl Munch Tierarztl Wochenschr
149	Neglected arthropod-borne viral infections in the Czech Republic	Review.	Epidemiol Mikrobiol Imunol
150	Why arboviruses can be neglected tropical diseases	Non-ILHV.	PLoS Negl Trop Dis
151	Outbreak of Chikungunya virus in the Indian Ocean: comments on a neglected infection	Non-ILHV.	Virologic
152	Recent advancement in flavivirus vaccine development	Review.	Expert Rev Vaccines

153	The worldwide challenges of "new" or reemerging communicable diseases at the dawn of the 21st century	Review.	Ann Pharm Fr
154	Persistence of arboviruses and antiviral antibodies in vertebrate hosts: its occurrence and impacts	Review.	Rev Med Virol
155	Present status of an arbovirus infection: yellow fever, its natural history of hemorrhagic fever, Rift Valley fever	Non-ILHV.	Bull Soc Pathol Exot
156	Tick-transmitted arbovirus in Maghreb	Non-ILHV.	Bull Soc Pathol Exot
157	The significance of mosquito longevity and blood-feeding behaviour in the dynamics of arbovirus infections	Non-ILHV.	Med biol
**SciELO**			
158	Study of Arboviruses in *Philander opossum*, *Didelphis marsupialis* and *Nectomys rattus* captured from forest fragments in the municipality of Belém, Pará, Brazil	Non-human.	Cienc Rural
159	Previous dengue or Zika virus exposure can drive to infection enhancement or neutralisation of other flaviviruses	DENV and ZIKV cross-react *in vitro*	Mem Inst Oswaldo Cruz
**160**	**Seroprevalence of flaviviruses antibodies in water buffaloes *(Bubalus bubalis)* in Brazilian Amazon**	**Non-human.**	**J Venon Anim Toxins incl Trop Dis**
**161**	**Neutralising antibodies for West Nile virus in horses from Brazilian Pantanal**	**Non-human.**	**Mem Inst Oswaldo Cruz**
162	Molecular characterization of two rocio flavivirus strains isolated during the encephalitis epidemic in são paulo state, brazil and the development of a one-step rt-pcr assay for diagnosis	Molecular characterization of flavivirus strains	Rev Inst Med trop S Paulo
163	Serologic survey for yellow fever and other arboviruses among inhabitants of Rio Branco, Brazil, before and three months after receiving the yellow fever 17D vaccine		Rev Soc Bras Med Trop
164	Brazilian Flavivirus phylogeny based on NS5	Flavivirus phylogeny	Mem Inst Oswaldo Cruz
165	Ilheus arbovirus in wild birds (Sporophila caerulescens and Molothrus bonariensis)	Non-human.	Rev Saúde Pública
166	Serological survey on arbovirus infection in residents of ecological reserve		Rev Saúde Pública
167	Surveillance of arbovirus infections in the atlantic forest region, State of São Paulo, Brazil: I. detection of hemagglutination-inhibition antibodies in wild birds between 1978 and 1990	Non-human.	Rev Inst Med trop S Paulo
**168**	**Research of antibodies to arbovirus in the serum of residentes of the village of Corte de Pedra, Valença, Bahia**		**Mem Inst Oswaldo Cruz**
**Science Direct**			
**169**	**Detection and characterization of Ilheus and Iguape virus genomes in historical mosquito samples from Southern Brazil**	**Non-human.**	**Acta Tropica**
170	Arbovirus outbreak in a rural region of the Brazilian Amazon	Non-ILHV.	J Clin Virol
171	Neurological infection by chikungunya and a triple Arbovirus co-infection in Mato Grosso, Central Western Brazil during 2019	Non-ILHV.	J Clin Virol
172	Insect-specific viruses and arboviruses in adult male culicids from Midwestern Brazil	Non-ILHV. Non-human.	Infect, Gen, Evol
173	Arbovirus investigation in patients from Mato Grosso during Zika and Chikungunya virus introdution in Brazil, 2015–2016	Non-ILHV. Non-human.	Acta Tropica
174	Ecological aspects of potential arbovirus vectors (Diptera: Culicidae) in an urban landscape of Southern Amazon, Brazil	Non-ILHV.	Acta Tropica
175	Reliable detection of St. Louis encephalitis virus by RT-nested PCR	Non-ILHV.	Enfer Infec Microbiol Clin
176	Arbovirus Encephalitis	Review.	Encyc Neurol Scienc
177	Le virus West Nile, un arbovirus émergentWest Nile virus, an emerging arbovirus	Non-ILHV.	La Presse Médicale
178	The virome of vector mosquitoes	Review.	Curr Opinion Virol

179	Arbovirus infections	Review.	Manson's Trop Infec Dis
180	Review of -omics studies on mosquito-borne viruses of the Flavivirus genus	Review.	Virus Res
181	Flaviviruses and where the Zika virus fits in: An overview	Review.	Neuroscience Zika
182	Arbovirus infections	Review.	Manson's Trop Infec Dis
183	Brain and Central Nervous System Infections: Viruses	Review.	Encyclopedia Infec Immun
184	Into the woods: Changes in mosquito community composition and presence of key vectors at increasing distances from the urban edge in urban forest parks in Manaus, Brazil	Non-ILHV. Non-human.	Acta Tropica
185	Zika virus in Thailand	Review.	Microb Infec
186	West Nile Virus in the Americas	Review.	Med Clin North America
187	Come fly with me: Review of clinically important arboviruses for global travelers	Review.	J Clin Virol
188	West Nile Virus (Flaviviridae)	Review.	Reference Module Biomed Sci
189	Arbovirus	Review.	Int Encyclopedia Public Health
190	The triple epidemics of arboviruses in Feira de Santana, Brazilian Northeast: Epidemiological characteristics and diffusion patterns	Non-ILHV.	Epidemics
191	Isolation of a novel insect-specific flavivirus with immunomodulatory effects in vertebrate systems	Non-ILHV.	Virology
192	Isolation and genomic characterization of Chaoyang virus strain ROK144 from *Aedes vexans nipponii* from the Republic of Korea	Non-ILHV.	Virology
193	Diversity of mosquito (Diptera: Culicidae) vectors in a heterogeneous landscape endemic for arboviruses	Non-ILHV. Non-human.	Acta Tropica
194	Mosquito-transmitted viruses – the great Brazilian challenge	Review.	Braz J Microbiol
195	Impact of alphavirus 3'UTR plasticity on mosquito transmission	Review.	Seminars Cell Develop Biol
196	Mosquitoes of British Honduras, with some comments on malaria, and on arbovirus antibodies in man and equines	Non-human.	Trans Royal Soc Trop Med Hyg
197	*Callithrix penicillata*: A feasible experimental model for dengue virus infection	Non-ILHV. Non-human.	Immunol Letters
198	Nonlocal dispersal of dengue in the state of Bahia	Non-ILHV. Non-human.	Scienc Total Enviro
199	Virus del Zika Enfrentarse a una nueva amenaza	Zika virus. Non-ILHV	Nursing
200	Arbovirus diseases	Review.	Synopsis Infec Trop Dis
201	Ecoepidemiology of *Alphaviruses* and *Flaviviruses*	Non-ILHV.	Emerg Reemerg Viral Pathog
202	Zika virus infection—the next wave after dengue?	Review.	J Formosan Med Assoc
203	Taxonomy of the virus family *Flaviviridae*	Non-ILHV.	Advances Virus Res
204	Molecular, ecological, and behavioural drivers of the bat-virus relationship	Review.	iScience
205	Animals as potential reservoirs for dengue transmission: A systematic review	Review.	One Health
206	Viral emergence and immune interplay in flavivirus vaccines	Non-ILHV.	Lancet Infect Dis
207	Simultaneous infection by DENV-3 and SLEV in Brazil	Non-ILHV.	J Clin Virol
208	Japanese Encephalitis, West Nile, and Other Flavivirus Infections	Review.	Trop Infect Dis: Principles, Pathog Practice

209	Come fly with me: Review of clinically important arboviruses for global travelers	Non-ILHV.	J Clin Virol
210	Virus Infections of the Central Nervous System	Review.	Manson's Trop Dis
211	Production of recombinant NS1 protein and its possible use in encephalitic flavivirus differential diagnosis	Non-ILHV.	Protein Expres Purif
212	Pathogenic flaviviruses	Review.	The Lancet
213	Undifferentiated tropical febrile illness in Cordoba, Colombia: Not everything is dengue	Non-ILHV.	J Infec Public Health
214	Analysis of a Reverse Transcription Loop-mediated Isothermal Amplification (RT-LAMP) for yellow fever diagnostic	Non-ILHV.	J Virol Methods
215	Epidemic in southern mexico of disease resembling virus hæmorrhagic fevers	Non-ILHV.	The Lancete
216	Kadam virus: Neutralization studies and laboratory transmission by *dermacentor variabilis*	Non-ILHV.	Trans Royal Soc Trop Med Hyg
217	The reintroduction of DENV-2 in 2011 in Panama and subsequent outbreak characteristic	Non-ILHV.	Acta Tropica
218	West Nile Virus: Epidemiology and Ecology in North America	Non-ILHV.	Advances Virus Res
219	Les flavivirus responsables de fièvres hémorragiques	Non-ILHV.	Annales de I'institut Pasteur
220	Absence of antigenic cross-reactions between rubella virus and arboviruses	Non-ILHV.	Virology
221	Detection of Culex flavivirus and Aedes flavivirus nucleotide sequences in mosquitoes from parks in the city of São Paulo, Brazil	Non-ILHV. Non-human.	Acta Tropica
222	Identification of Simbu, California and Bunyamwera serogroup bunyaviruses by nested RT-PCR	Non-ILHV.	Trans Royal Soc Trop Med Hyg
223	Arboviruses: Incorporation in a General System of Virus Classification	Review.	Comparative Virology
224	Arboviruses, Encephalitis Caused by	Review.	Ency Neurol Scien
225	Mosquito-borne diseases	Review.	Dengue Virus Dis
226	Arboviruses: alphaviruses, flaviviruses and bunyaviruses: Encephalitis; yellow fever; dengue; haemorrhagic fever; miscellaneous tropical fevers; undifferentiated fever	Review.	Med Microbiol
227	Viral encephalitis: familiar infections and emerging pathogens	Review.	The Lancet
228	Flaviviruses	Review.	Princ Pratic Pediatric Infect Dis
229	A virus disease of the malaria parasite	Non-ILHV.	Trans Royal Soc Trop Med Hyg
230	Neuropathology of S. Paulo south coast epidemic encephalitis (*Rocio flavivirus*)	Confusing laboratory diagnosis	J Neurol Scien
231	Flaviviruses	Review.	Princ Pratic Pediatric Infect Dis
232	Flaviviridae	Review.	Fenner's Veterinary Virology
233	The first isolation of bussuquara virus from man	Non-ILHV.	Trans Royal Soc Trop Med Hyg
234	The arboviruses	Non-ILHV.	Introduction General Virology
235	Enzyme immunoassay for the detection of dengue IgG and IgM antibodies using infected mosquito cells as antigen	Non-ILHV.	Trans Royal Soc Trop Med Hyg
236	Viral Diseases	Review.	Neuroepidemiol Trop Health
237	Flavivirus Encephalitides	Review.	Equine Infect Dis

238	Mosquitoes (Culicidae)	Review.	Med Veter Entomol
239	The need for chemotherapy and prophylaxis against viral diseases	Review.	Perspectives Med Virol
240	Evolution, epidemiology, and dispersal of flaviviruses revealed by molecular phylogenies	Review.	Advances Virus Research
241	The Positive Sense Single Stranded RNA Viruses	Review.	Virus Taxonomy
242	Immunosuppression And Experimental Virus Infection Of The Nervous System	Non-ILHV.	Advances Virus Research
243	Serodiagnosis of Flaviviral Infections and Vaccinations in Humans	Non-ILHV.	Advances Virus Research
244	Mosquitoes (culicidae)	Non-ILHV.	Med Vet Entomology
245	Mosquitoes and The Incidence of Encephalitis	Non-ILHV.	Advances Virus Research
246	Genetics of Resistance of Animals to Viruses: I. Introduction and Studies in Mice	Non-ILHV.	Advances Virus Research
247	The History and Evolution of Human Dengue Emergence	Review.	Advances Virus Research
248	Molecular Amplification Assays for the Detection of Flaviviruses	Review.	Advances Virus Research
249	Medical Acarology and Entomology	Non-ILHV.	Manson's Trop Infect Dis
250	Epidemiology of Arthropod-Borne Togaviruses: The Role of Arthropods as Hosts and Vectors and of Vertebrate Hosts in Natural Transmission Cycles	Review.	Togaviruses
251	Virus research	Review	Non-human Primates Med Res
**Adittional records**			
252	Crystal structure of the Ilheus virus helicase: implications for enzyme function and drug design	In silico study.	Cell Biosci
253	Prevalence of arbovirus antibodies in young healthy adult population in Brazil		Parasit Vectors
254	Serologic evidence of West Nile virus and Saint Louis encephalitis virus in horses from Southern Brazil	Non-human.	Braz J Microbiol
255	Individual, household and environmental factors associated with arboviruses in rural human populations, Brazil		Zoonoses Public Health
256	Insect-specific viruses and arboviruses in adult male culicids from Midwestern Brazil	Non-human.	Infect Genet Evol
257	Fatal Outcome of Ilheus Virus in the Cerebrospinal Fluid of a Patient Diagnosed with Encephalitis		Viruses
258	Applying a pan-flavivirus RT-qPCR assay in Brazilian public health surveillance	Non-human.	Arch Virol
259	Detection and characterization of Ilheus and Iguape virus genomes in historical mosquito samples from Southern Brazil	Non-human.	Acta Tropica
260	Investigation about the Occurrence of Transmission Cycles of Arbovirus in the Tropical Forest, Amazon Region	Non-human.	Viruses
261	Detection of Ilheus virus in mosquitoes from southeast Amazon, Brazil	Non-human.	Trans R Soc Trop Med Hyg
262	Detection of antibodies against Icoaraci, Ilhéus, and Saint Louis Encephalitis arboviruses during yellow fever monitoring surveillance in non-human primates (Alouatta caraya) in southern Brazil	Non-human.	J Med Primatol
263	Ilheus and Saint Louis encephalitis viruses elicit cross-protection against a lethal Rocio virus challenge in mice	Non-human.	PLoS One
264	Detection of the mosquito-borne flaviviruses, West Nile, Dengue, Saint Louis Encephalitis, Ilheus, Bussuquara, and Yellow Fever in free-ranging black howlers (Alouatta caraya) of Northeastern Argentina	Non-human.	PLoS Negl Trop Dis
265	Serosurvey of selected arboviral pathogens in free-ranging, two-toed sloths (choloepus hoffmanni) and three-toed sloths (bradypus variegatus) in costa rica, 2005-07	Non-human.	J Wild Dis

266	Seroprevalence of flaviviruses antibodies in water buffaloes (Bubalus bubalis) in Brazilian Amazon	Non-human.	J Venom Anim Toxins Incl Trop Dis
267	Serological Evidence of Widespread Circulation of West Nile Virus and Other Flaviviruses in Equines of the Pantanal, Brazil	Non-human.	PLoS Negl Trop Dis
268	Ilheus virus isolation in the Pantanal, west-central Brazil	Non-human.	PLoS Negl Trop Dis
269	Ilheus virus infection in human, Bolivia		Emerg Infec Dis
270	Neutralising antibodies for West Nile virus in horses from Brazilian Pantanal	Non-human.	Mem Inst Oswaldo Cruz
271	New records and epidemiological potential of certain species of mosquito (Diptera, Culicidae) in the State of Rio Grande do Sul, Brazil	Non-human.	Rev Soc Bras Med Trop
272	Etiology of acute undifferentiated febrile illness in the Amazon basin of Ecuador		Am J Trop Med Hyg
273	West Nile, Ilheus, and Bunyamwera Virus Infections in Man 1,2,3		Am J Trop Med Hyg
274	Arthropod-borne virus antibodies in sera from residents of South-East Asia		Trans R Soc Trop Med Hyg
275	Virus antibody survey on sera of residents of the Amazon valley in Brazil*	Data not available.	Am J Trop Med Hyg
276	Neutralizing antibodies against certain viruses in the sera of residents of Trinidad, B. W. I	Data not available.	Am J Trop Med Hyg
277	Isolation of Ilheus virus from human beings in Trinidad, west indies		Trans R Soc Trop Med Hyg
278	Report of a 1958 Outbreak in Miami and a Serologic Survey of Miami Residents		Am J Med
279	The isolation of arthropod-borne viruses, including members of two hitherto undescribed serological groups, in the Amazon region of Brazil		Am J Trop Med Hyg
280	Neutralizing and haemagglutination-inhibiting antibodies to yellow fever 17 years after vaccination with 17D vaccine*		Bull World Health Organ
281	Ecological Observations on Ilhéus Virus in the Vicinity of Almirante, Republic of Panama		Am J Trop Med Hyg
282	Estudios Epidemiológicos sobre virus arbor en el Sureste de México		Salud Publica Mex
283	Further studies of the Xavante indians		Am J Trop Med Hyg
284	A nationwide serum survey of Brazilian military recruits, 1964: II. Antibody patterns with arboviruses, polioviruses, measles and mumps		Am J Epidemiol
285	The Isolation of Ilhéus Virus from Man in Panamá		Am J Trop Med Hyg
286	Arthropod-Borne Encephalitis Viruses in Northeastern South America	Data not available.	Trop Geogr Med
287	Arbovirus neutralization tests with Peruvian sera in Vero cell cultures		Bull World Health Organ
288	Arbovirus antibody survey of sera from residents of eastern Peru		PAHO BULLETIN
289	Prevalence of antibody against viruses in the Tiriyo, an isolated amazon tribe		Am J Epidemiol
290	Isolation of Ilhéus Virus from Man in Colombia*		Am J Trop Med Hyg
291	A nationwide serum survey of Colombian military recruits, 1966 I. Description of sample and antibody patterns with arboviruses, polioviruses, respiratory viruses, tetanus, and treponematosis		Am J Epidemiol
292	Encuesta serologica de virus transmitidos por artropodos		Bol Oficina Sanit Panam
293	Serological survey for flavivirus in the human population of Zulia State, Venezuela, in 1967	Data not available.	Investigacion Clinica
294	Arbovirus antibodies in children of rural Guanabara, Brazil		Intervirol
295	Infectious diseases along Brazil's Trans-Amazon highway: surveillance and research		PAHO BULLETIN
296	Surveillance and research on infectious diseases along the trans-Amazon highway		Bull Pan Am Health Organ
297	Ecology of Arboviruses and their diseases in French Guiana		PAHO BULLETIN

298	Serologic survey of dengue and other arboviruses in Curaçao and Aruba, 1973		Bull Pan Am Health Organ
299	Seroepidemiologic studies in Oaxaca, Mexico. II. Survey for arbovirus antibody		Arch Invest Med
300	A multidisciplinary program of infectious disease surveillance along the Transamazon highway in Brazil: epidemiology of arbovirus infections		Bull Pan Am Health Organ
301	Modifications of arbovirus transmission in relation to construction of dams in Brazilian Amazonia		J Braz Asso Adv Scien
302	Estudos sorológicos para pesquisa de anticorpos de arbovírus em população humana da região do Vale do Ribeira III inquérito em coabitantes com casos de encefalite por Flavivirus Rocio		Rev Saude Publica
303	A serological study for research of arbovirus antibodies in human population of the Ribeira Valley Region. II – A survey of patients in the Pariquera-Açú Regional Hospital, 1980		Rev Saude Publica
304	Research of antibodies to arbovirus in the serum of residentes of the village of Corte de Pedra, Valença, Bahia		Mem Inst Oswaldo Cruz
305	Arbovirus antibody levels in the population of the Ribeirão Preto area, S.Paulo State (Brazil)		Rev Saude Publica
306	Isolation of Ilheus virus in French Guyana.		Bulletin de la Societe de Pathologie
307	Human disease caused by an arbovirus closely related to Ilheus virus: report of five cases		Intervirology
308	Ilheus virus isolate from a human, Ecuador.		Emerg Infect Dis

S5 File: PRISMA 2009 checklist showing guidelines for systematic reviews and meta-analysis

For more information, visit: **www.prisma-statement.org**.

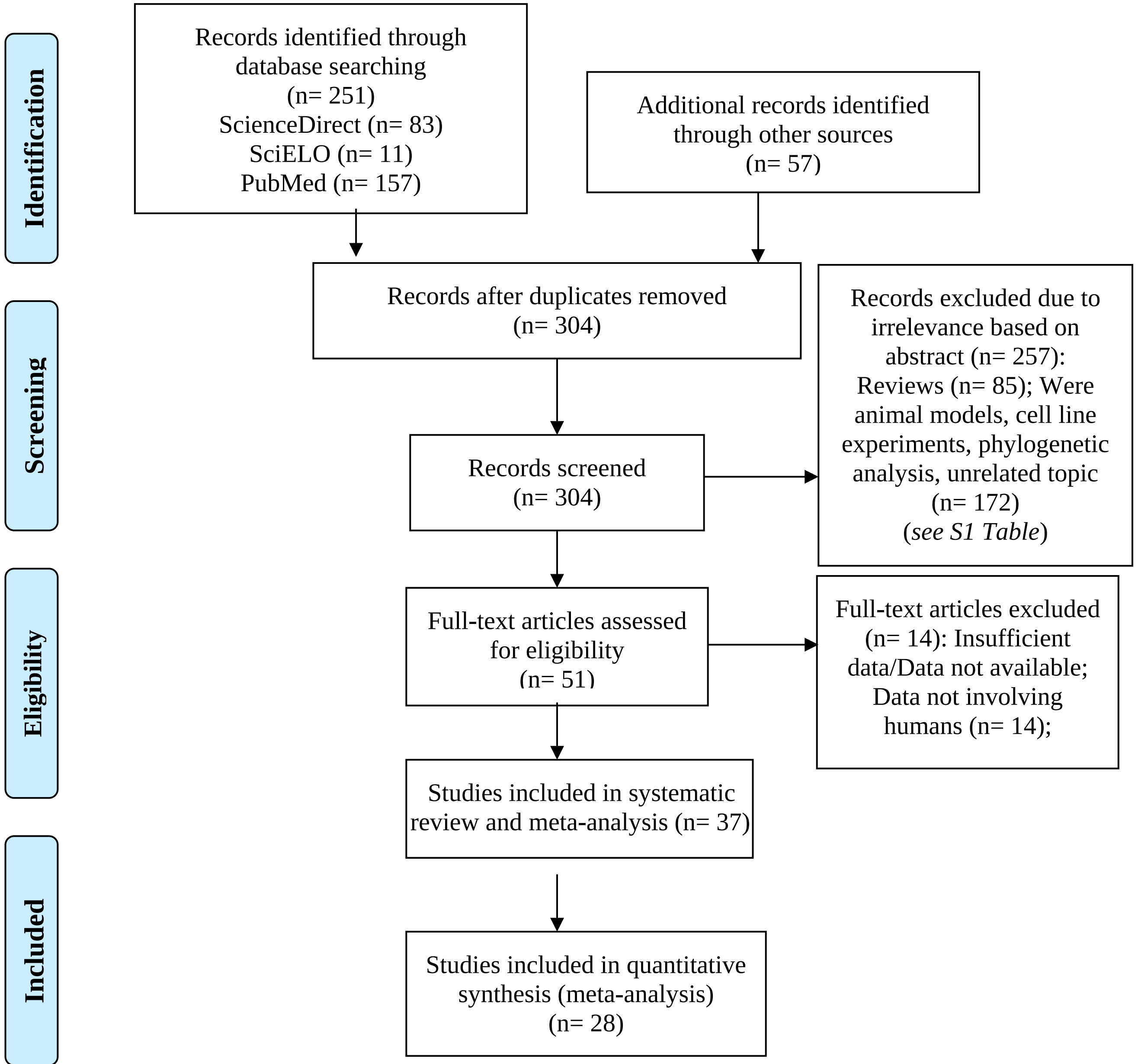

S6 File. The characteristics of the studies included in the systematic review and meta-analysis

	ID[1]	Biological material collected	Total humans/specimens	(%) ILHV's positivity	Diagnostic method	Suspected to have ILH fever?	Clinical arbovirus infection (sign/symptom)	Study period	Place of study
1	Southam et al 1951. West Nile, Ilheus, and Bunyamwera Virus Infections in Man 1,2,3	Blood samples/serum	19	100		Yes	Febrile persons	1951	USA
2	Prías-Landínez et al 1966. Encuesta serologica de virus transmitidos por artropodos.	Blood samples/serum	396	19.9	HI/NT	-	-	1966	Araracuara, Colombia
3	Prías-Landínez, et al 1968. Isolation of Ilhéus Virus from Man in Colombia*		1	100	HI/T	Yes	Febrile person	1968	Colombia
4	Pinheiro et al 1975. Arbovirus antibodies in children of rural Guanabara, Brazil.	Blood samples/serum	267	0.7	HI/NT	-	-	1968	Guanabara, Brazil
5	Pinheiro et al 1974. Infectious diseases along Brazil's trans-amazon highway: surveillance and research.	Blood samples/serum	308	2.2	HI	Yes	Febrile persons	1971-72	Altamira, Brazil
6	Weiland et al 1978. Serologic survey of dengue and other arboviruses in Curaçao and Aruba, 1973.	Blood samples/serum	3,044	3.9	HI	-	-	1973	Curaçao and Aruba
7	Dixon et al 1981. A multidisciplinary program of infectious disease surveillance along the transamazon highway in Brazil: epidemiology of arbovirus infections	Blood samples/serum	1,603	2.3	HI	-	-	1974	Marabá and Altamira, Brazil
8	Dégallier et al 1992. Modifications of arbovirus transmission in relation to construction of dams in Brazilian Amazonia	Blood samples/serum	1,940	18.2	HI or NT	-	-	1982-88	Tucuruí and Altamira, Brazil
9	Iversson et al 1982. Estudos sorológicos para pesquisa de anticorpos de arbovírus em população humana da região do vale ribeira.	Blood samples/serum	82	7.3	HI	-	Encephalitis	1975-78	Vale do Ribeira, Brazil
10	Iversson et al 1981. Estudos sorológicos para pesquisa de anticorpos de arbovírus em população humana da região do vale ribeira. II- Inquérito em pacientes do Hospital Regional de Pariquera-Açú, 1980.	Blood samples/serum	516	5.2	HI/NT	-	-	1980	Vale do Ribeira, Brazil
11	Tavares-Neto et al 1986. Pesquisa de anticorpos para arbovirus no soro de residentes no povoado de Corte de Pedra, Valença, Bahia.	Blood samples/serum	288	2.0	HI/NT/CF	-	-	1984	Valença, Brazil
12	Figueiredo et al 1986. Níveis de anticorpos para arbovírus em indivíduos de Ribeirão Preto, SP (Brasil).	Blood samples/serum	302	1.3	HI/NT/CF	-	-	-	Ribeirão Preto, Brazil
13	Nassar et al 1997. Human disease caused by an arbovirus closely related to Ilheus virus: report of five cases.	Blood samples/serum	5	100	Isolated virus/HI/NT/CF	Yes	Fever, headache, myalgia, arthralgia	1995	São Paulo, Brazil
14	Straatmann et al 1997. Evidências sorológicas da circulação do arbovírus Rocio (Flaviviridae) na Bahia.	Blood samples/serum	689	0.29	HI/NT	-	-	1995	Bahia, Brazil

15	Araújo et al 2002. Serological diagnosis of dengue and yellow fever infections in suspected cases from Pará State, Brazil, 1999.	Blood samples/serum	785	0.7	HI/ELISA	-	-	1999	Pará, Brazil
16	Cruz et al 2009. Serological survey for arboviruses in Juruti, Pará State, Brazil.	Blood samples/serum	1,597	0.9	HI	-	-	2007-08	Pará, Brazil
17	Catenacci et al 2021. Individual, household and environmental factors associated with arboviruses in rural human populations, Brazil.	Blood samples/serum	523	61.9	HI	-	-	-	Bahia, Brazil
18	Tavares-Neto et al 2004. Serologic survey for yellow fever and other arboviruses among inhabitants of Rio Branco, Brazil, before and three months after receiving the yellow fever 17D vaccine.	Blood samples/serum	390	5.9	HI	-	-	1999	Acre, Brazil
19	Salgado et al 2021. Prevalence of arbovirus antibodies in young healthy adult population in Brazil.	Blood samples/serum	298	51.3	HI	-	-	2014-15	Amazonas, Brazil
20	Ehrenkranz et al 1963. Arthropod-borne virus disease in Florida. Report of a 1958 outbreak in Miami and a serological survey of Miami Residents.	Blood samples/serum	18	0	HI	Yes	Meningitits or encephalitis	1958	Florida, USA
21	Black et al 1970. Prevalence of antibody against viruses in the Tiriyo, and isolated Amazon tribe.	Blood samples/serum	178	4.4	HI	-	-	1966	Brazil-Surinam border
22	Evans et al 1969. A nationwide serum survey of Colombian military recruits, 1966. I. Description of samples and antibody patterns with arboviruses, polioviruses, respiratory viruses, tetanus, and treponematosis.	Blood samples/serum	292	3.7	HI	-	-	1966	Columbia
23	Groot et al 1962. Neutralizing and haemagglutination-inhibiting antibodies to yellow fever 17 years after vaccination with 17D vaccine.	Blood samples/serum	182	14.8	HI	-	-	1941/58	Minas Gerais, Brazil
24	Macías 1963. Estudios epidemiologicos sobre virus arbor en el sureste de Mexico.	Blood samples/serum	215	9.3	HI	-	-	1962	Veracruz, Mexico
25	Niederman et al 1967. A nationwide serum survey of Brazilian military recruits, 1964. Antibody patterns with arboviruses, polioviruses, measles and mumps.	Blood samples/serum	1,037	16.2	HI	-	-	1964	Brazil
26	Rodaniche and Galindo 1963. Ecological observations on Ilhéus virus in the vicinity of Almirante, republic of Panama.	Blood samples/serum	643	14.9	NT	-	-	1960	Panama
27	Madalengoitia et al 1973. Arbovirus antibody survey of sera from residents of eastern, Peru.	Blood samples/serum	1,063	17.5	HI	-	-	1965	Peru
28	Buckley et al 1972. Arbovirus neutralization tests with Peruvian sera in Vero cell cultures.	Blood samples/serum	89	65.1	HI/NT	-	-	-	Peru
29	Romano-Lieber et al 2000. Serological survey on arbovirus infection on residents of ecological reserve.	Blood samples/serum	182	2.2	HI	-	-	1990	Vale do Ribeira, Brazil
30	Neél et al 1968. Further studies of the Xavante indians.	Blood samples/serum	412	18.9	HI			-	Mato Grosso, Brazil
31	Milhim et al 2020. Fatal outcome of Ilheus virus in the cerebrospinal fluid of a patient diagnosed with encephalitis.	Cerebrospinal fluid	287	0.3	PCR assay	-	Encephalitis	2016-2017	São Paulo, Brazil
32	Causey et al 1961. The isolation of arthropod-borne virus, including members of two hitherto undescribed serological groups, in the Amazon region of Brazil.		2				Undifferentiated Febrile		Amazon, Brazil

33	Venegas et al 2012. Ilheus virus infection in Human, Bolivia.	Blood samples/serum	1	100	IgM ELISA / PCR assay	-	Fever, headache, arthralgia...	2005	Bolivia
34	Johnson et al 2007. Ilheus virus isolate from a human, Ecuador.	Blood samples/serum	1	100	Virus isolation/PCR assay	Yes	Fever, rash, headache, arthralgia...	2004	Ecuador
35	Manock et al 2009. Etiology of acute undifferentiated febrile illness in the Amazon basin of Ecuador.	Blood samples/serum	11	27.2	IgM ELISA		Undifferentiated Febrile	2001-04	Ecuador
36	Srihongse and Johnson 1967. The isolation of Ilhéus virus from man in Panamá.	Blood samples/serum	348	27	HI	-	-	1964	Panama
37	Spence et al 1962. Isolation of Ilhéus virus from human beings in Trinidad, West indies.		3	100	Virus isolation		Fever, headache, arthralgia...	1955-57	Trinidad

[1]ID: Identification of the study;

S7 File. Methodologic quality of cohort and case-control studies included in the meta-analysis

Methodologic quality of cohort studies included in the meta-analysis (NOS)

The “star” presents a “high-quality” choice of individual study. For high-quality study was defined as a study with ≥4 awarded stars.

Methodologic quality of case-control studies included in the meta-analysis (Newcastle-Ottawa scale (NOS))

Study and year	Representa-tiveness of cases	Selection of non-exposed	Ascertainment of exposure	Demonstration outcome	Assessment of outcome	Follow-up outcomes	Adequacy of follow-up	Total quality scores
Groot et 1962	☆	☆	–	☆	☆	–	-	4
Ehrenkranz et al 1963	☆	-		☆	☆		-	3
Macías et al 1963	☆	☆	-	☆	–	-	-	3
Rodaniche et al 1963	☆	☆	-	☆	–	-	-	3
Prías-Landínez et al 1966	☆	☆	–	☆	☆		-	4
Neél et al 1967	☆	☆	-	☆	–	-	-	3
Niederman et al 1967	☆	-	-	☆	☆	-	-	3
Srihongse et al 1967	☆	☆	-	☆	–	-	-	3
Evans et al 1969	☆	-	–	☆	☆		-	3
Black et al 1970	☆	☆	-	☆	☆	-	-	4
Buckley et al 1972	☆	☆	-	☆	–	-	-	3
Madalengoitia et al 1973	☆	-	-	☆	☆	-	-	3
Pinheiro et al 1974	☆	☆	-	☆	☆	-	-	4
Pinheiro et al 1975	☆	☆	-	☆	☆	-	-	4
Weiland et al 1978	☆	☆	-	☆	☆	-	-	4
Dixon et al 1981	☆	☆		☆	☆		-	4
Iversson et al 1981	☆	☆	-	☆	☆	-	-	4
Iversson et al 1982	☆	☆	–	☆	☆	-	-	4
Figueiredo et al 1986	☆	☆	-	☆	☆		-	4

Tavares-Neto et al 1986	☆	☆	-	☆	☆	-	-	4
Dégallier et al 1992	☆	☆	–	☆	–	-	-	3
Straatmann et al 1997	☆	☆	-	☆	–	-	-	3
Romano-L. et al 2000	☆	☆	-	☆	☆	-	-	4
Araújo et al 2002	☆	☆	-	☆	☆	-	-	4
Tavares-Neto et al 2004	☆	☆	-	☆	☆	-	-	4
Cruz et al 2009	☆	☆	-	☆	☆	-	-	4
Salgado et al 2021	☆	☆	☆	☆	☆		-	5
Catenacci et al 2021	☆	☆	☆	☆	☆		-	5

S8 File. Sensitivity analysis for the effect of removing one study at a time on the overall estimation of ILHV positivity in serological reactions of heterotypic (A) and monotypic (B) type.

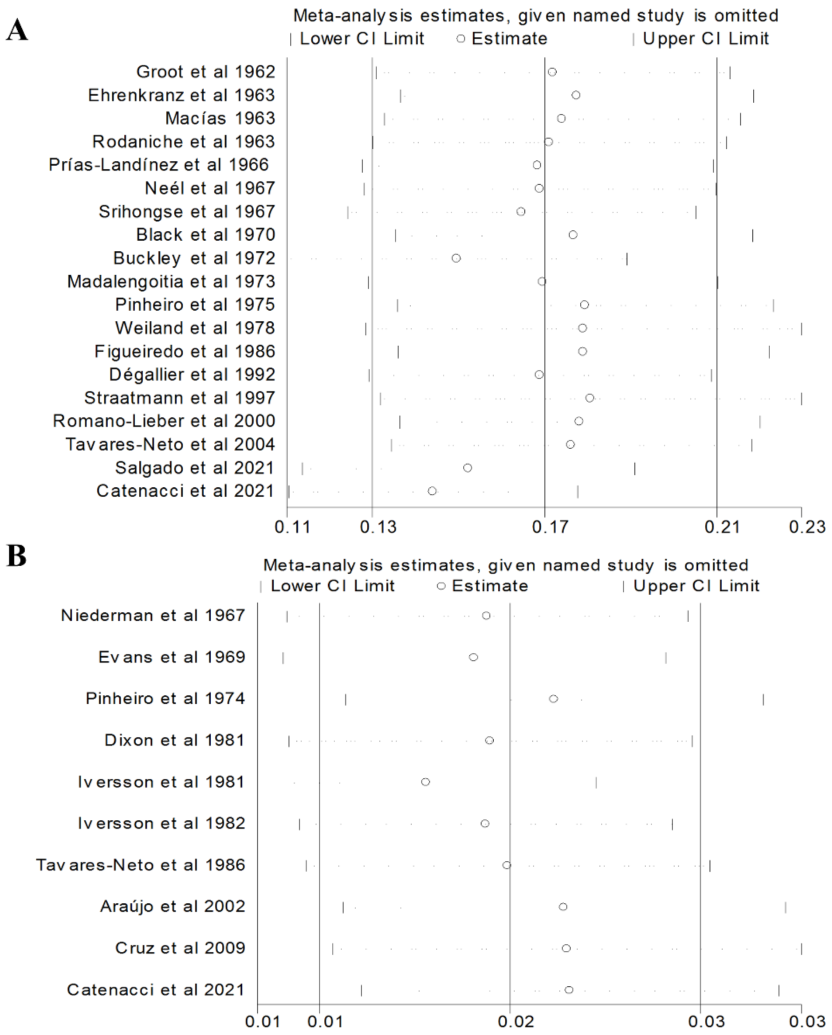

S9 File. Forest plot of the proportion of laboratory confirmed ILHV infection according the regions of origin of the articles

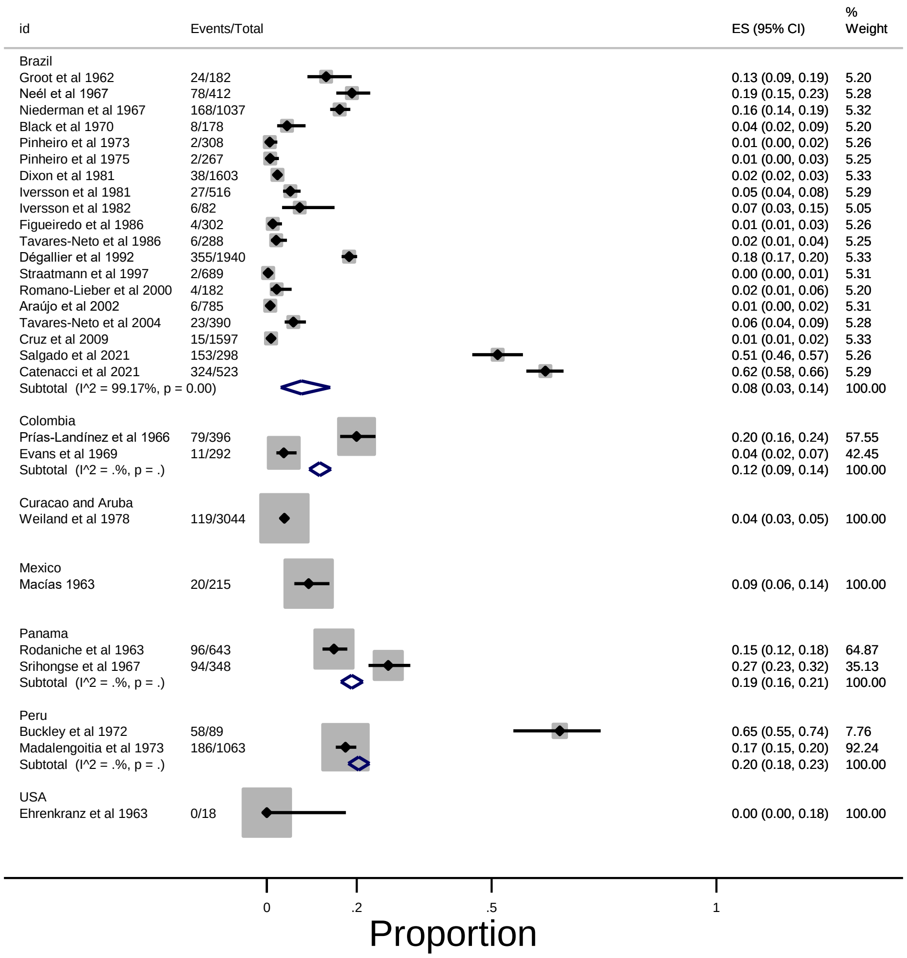

S10 File. Forest plot of the proportion of laboratory confirmed ILHV infection according the age of individuals.

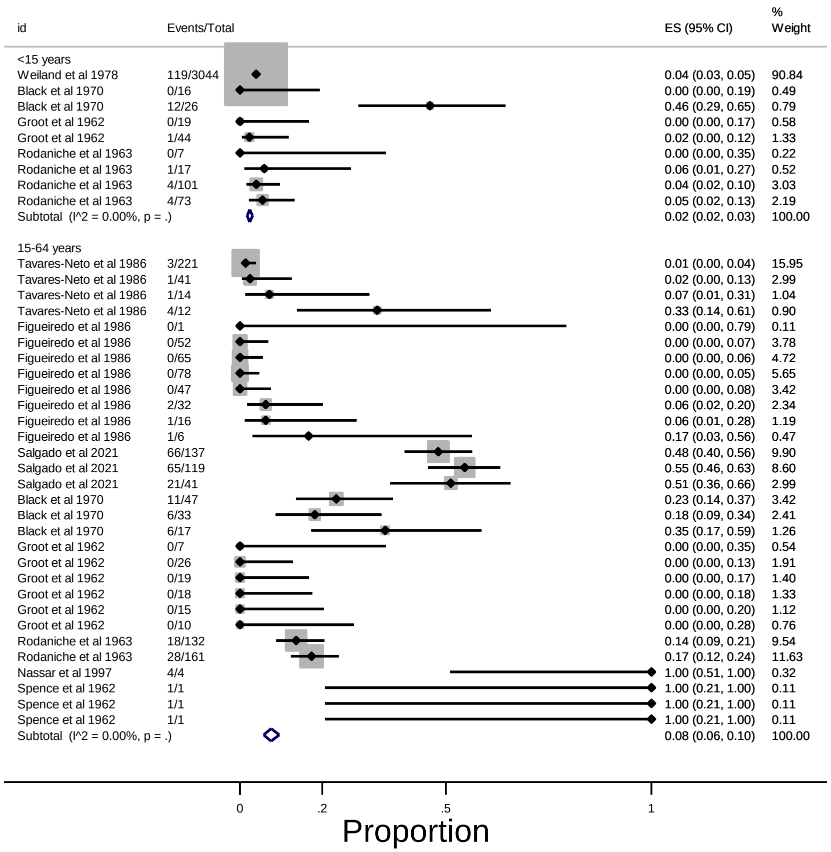

S11 File. Forest plot of the odds ratio for laboratory confirmed ILHV infection relating to the sex of individuals

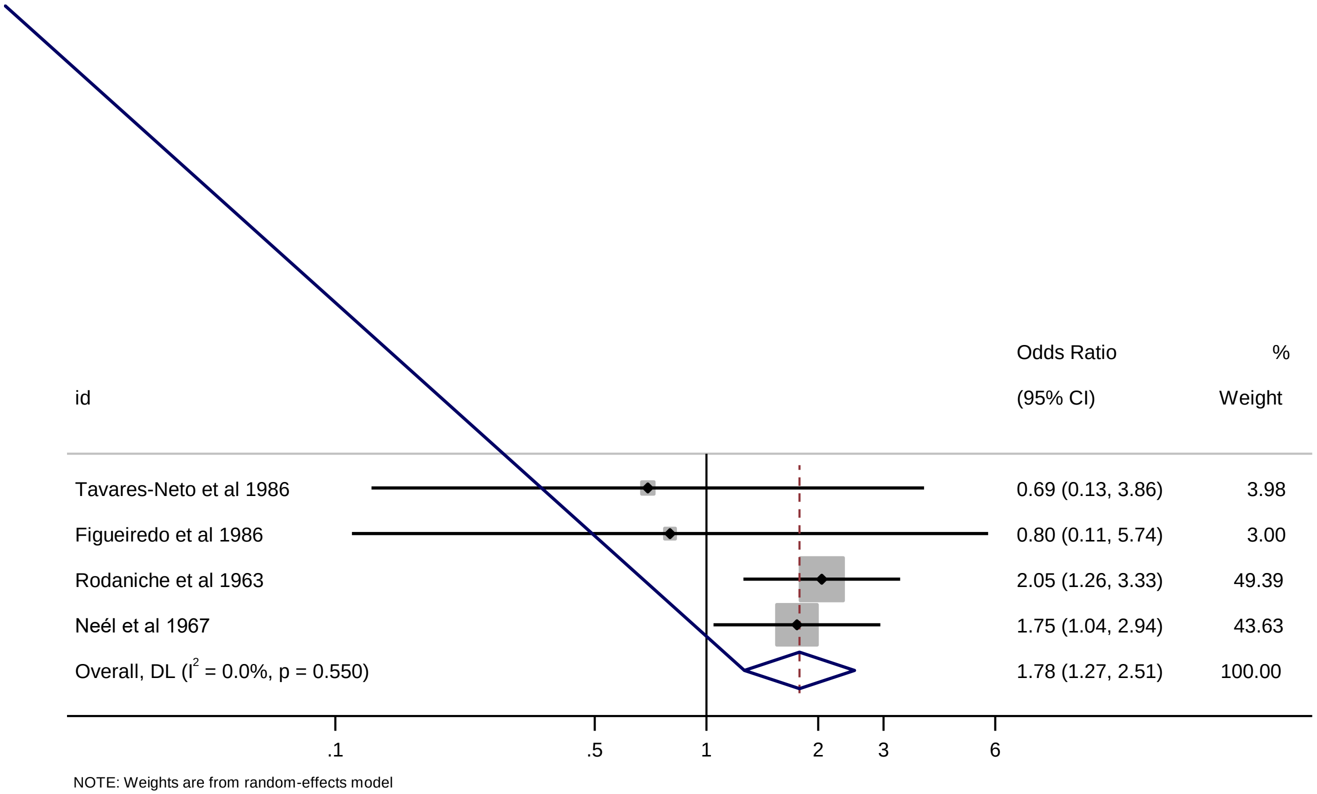

Odds Ratio
%
id
(95% CI)
Weight
Tavares-Neto et al 1986
0.69 (0.13, 3.86)
3.98
Figueiredo et al 1986
0.80 (0.11, 5.74)
3.00
Rodaniche et al 1963
2.05 (1.26, 3.33)
49.39
Neél et al 1967
1.75 (1.04, 2.94)
43.63
Overall, DL (I2 = 0.0%, p = 0.550)
1.78 (1.27, 2.51)
100.00
.1
.5
1
2
3
6
NOTE: Weights are from random-effects model
